# Identifying bureaus with substantial personnel change during the Trump administration: A Bayesian approach

**DOI:** 10.1371/journal.pone.0278458

**Published:** 2023-01-18

**Authors:** Brian Libgober, Mark D. Richardson

**Affiliations:** 1 Department of Political Science, Northwestern University, Evanstan, Illinois, United States of America; 2 Department of Government, Georgetown University, Washington, District of Columbia, United States of America; Public Library of Science, UNITED STATES

## Abstract

Presidents and executive branch agencies often have adversarial relationships. Early accounts suggest that these antagonisms may have been deeper and broader under President Trump than under any recent President. Yet careful appraisals have sometimes shown that claims about what President Trump has done to government and politics are over-stated, require greater nuance, or are just plain wrong. In this article, we use federal employment records from the Office of Personnel Management to examine rates of entry and exit at agencies across the executive branch during President Trump’s term. A key challenge in this endeavor is that agencies vary in size dramatically, and this variability makes direct comparisons of rates of entry and exit across agencies problematic. Small agencies are overrepresented among agencies with large and small rates. Yet small agencies do important work and cannot simply be ignored. To address such small-area issues, we use a Bayesian hierarchical model to generate size-adjusted rates that better reflect the fundamental uncertainty about what is happening in small agencies as well as the substantial likelihood that these entities are less unusual than raw statistics imply. Our analysis of these adjusted rates leads to three key findings. First, total employment at the end of the Trump administration was largely unchanged from where it began in January of 2017. Second, this aggregate stability masks significant variation across departments, with immigration-focused bureaus and veterans-affairs bureaus growing significantly and certain civil-rights focused bureaus exhibiting signs of stress. Finally, compared to the first terms of Presidents Bush and Obama, separation rates under President Trump were markedly higher for most agencies.

## Personnel politics under the Trump administration

On September 1, 2019, President Trump tweeted that “one of the largest hurricanes ever” was approaching Florida, Georgia, the Carolinas, and Alabama and that these states “will most likely be hit (much) harder than anticipated” [1]. However, the most up-to-date forecasts by the National Weather Service did not include Alabama in the list of states at risk due to Hurricane Dorian. Twenty minutes later, responding to calls from distressed residents, the Birmingham Office of the National Weather Service tweeted that “Alabama will NOT see any impacts from Hurricane Dorian” [1]. Over the next several days, President Trump continued to insist that his forecast was correct, even presenting to reporters a weather map apparently doctored by a Sharpie marker [2]. Days later, the National Oceanic and Atmospheric Administration (NOAA), which contains the National Weather Service, released a statement saying that the Birmingham Office had spoken in terms that were “inconsistent with probabilities from the best forecasts” available at the time [1]. According to reporting from the *New York Times*, the statement was prompted by threats from Secretary of Commerce Wilbur Ross to fire top officials unless they fixed the “perceived contradiction of the President” [3].

Similarly tense episodes between the President and members of the civil service occurred with surprising frequency during the Trump-era. From US Ambassador to Ukraine Marie Yovanovitch [4], to the US Attorney in Manhattan Geoffrey Berman, to the lead election security official Chris Krebs, the Trump administration provoked numerous scandals through the firing of figures visibly frustrating the President’s agenda. Scores of less prominent officials have resigned in protest [5, 6], while others have sought greener pastures rather than persevere through such adversities. Watchdogs have pulled numerous fire alarms warning that agencies are being gutted by the Trump administration [7]. Trump loyalists did little to dispute these claims, instead arguing that they were part and parcel of Trump’s campaign promise to “drain the swamp.”

Many would assume that these examples are only the most glaring instances of Trump undermining the civil service [5]. Yet, there is also reason to believe otherwise. Trump may have done far less to erode the civil service than these stories suggest. Media acccounts are only the “first rough draft of history,” and later drafts may look quite different. For example, the Commerce Department’s Inspector General, an Obama appointee, later determined that while “it was reasonable under the circumstances” for some employees to infer that their jobs depended on issuing a statement deferential to the White House, no one was actually fired as a result of Sharpiegate. Even more shockingly, there was actually no “credible evidence that showed that Secretary Ross or anyone else directly threatened to fire. [any] Department or NOAA employee” [8]. There are many other examples of officials portrayed as working under the Sword of Damocles who did not ultimately lose their jobs, from Rosenstein to Mueller to Fauci and beyond.

These paradoxes of the Trump administration are not unique to its relationship with the civil service, but extend to many other domains, from regulation to party politics and beyond. A growing, inter-disciplinary literature has sought to evaluate many of the initial impressions about the impact of Trump, often with a view toward generalizable strategies for evaluating past and future administrations [9–13]. Given the importance of the civil service, across so many diverse areas, coming to an understanding of how the Trump-era impacted the federal workforce is particularly crucial.

In this article, we use publicly available federal employment records maintained by the Office of Personnel Management (OPM) to explore the Trump administration’s approach to personnel politics. We identify the bureaus and departments that have faced the most significant losses, gains, and churn in agency employment. A key methodological and interpretive challenge to such a task is that a naive analysis of raw hiring and departure rates will tend to overstate the significance of movements in small agencies. One solution is to restrict attention to the story as it pertains to large agencies [14], however it is ex ante plausible that smaller agencies are where the most significant action is. Our approach leverages Bayesian hierarchical models to add a principled amount of skepticism as to whether small agencies have faced employment pressures significantly different from the mean. We make available detailed estimates of attrition and hiring rates across three administrations that are useful for interested researchers and policymakers.

Our analysis yields three primary conclusions. First, the total number of non-seasonal full-time permanent career civil servants at the end of the Trump administration was near where it began in January of 2017, despite most departments losing employees. Second, this aggregate stability masks significant variation in growth rates across agencies. Growth was concentrated in the Department of Veterans Affairs with total employment at the Departments of Defense, Health and Human Services, Homeland Security, and Commerce largely stable (Health and Human Services grew slightly while Defense, Homeland Security, and Commerce shrank slightly). All other Departments experienced a net loss of employees. At the bureau-level, we find that several civil-rights focused offices shrank notably at the same time as they undertook substantial hiring, indicating a particularly high amount of churn for these bureaus. Additionally, we find that bureaus responsible for implementing Trump administration priorities related to immigration and veterans affairs grew significantly. Finally, compared to the first terms of the George W. Bush and Obama administrations, separation rates were markedly higher in the Trump administration.

## Data and methods

Since the 1970’s, the Office of Personnel Management has maintained a unified database of employment records on federal civil servants for nearly all federal agencies. (The most recent version of the data is available here: https://www.fedscope.opm.gov/). OPM makes two kinds of employment records publicly available. Status files are quarterly snapshots of who works for the federal government. In these tables, each row is a person and the columns detail their background and aspects of their employment. Dynamic files identify the beginning and end of employment for the federal government. OPM calls these events “accessions” and “separations,” respectively. Dynamic files are reported by month. In these tables, each row reflects an employee’s onboarding or departure. These tables also include information about the nature of the change in employment status. There is substantial, but not complete overlap in the aspects of employment provided on each civil servant between the status files and the dynamic files. To focus on the career civil servants who serve across administrations in the following analysis, we subset the data to only non-seasonal full-time permanent civil servants, excluding Schedule C appointees, non-career members of the Senior Executive Service, and employees on the Executive Schedule pay plan, who are largely appointees confirmed by the Senate. For example, Level I of the EX pay plan includes department secretaries. We also exclude accessions and separations that OPM designates as mass transfers because mass transfers occur when the employees’ job function is also transferred (e.g., the creation of the Department of Homeland Security in the George W. Bush administration). Therefore, it would not be appropriate to use mass transfers to make inferences about individual-level career decisions, which is our goal in this paper. Hereafter, we refer to subcomponents of executive departments as bureaus, regardless of whether they are formally described as an administration, office, or some other kind of entity. We refer to independent agencies, executive departments, and agencies in the Executive Office of the President as agencies, unless we are referring only to executive departments. To make this concrete, under this vernacular NOAA is a bureau and its parent, the Department of Commerce, is an agency. Refer to Section S1 of the S1 File for additional description of the OPM data and discussion of coding decisions.

### Model and assumptions

The individual career decisions that generate rates of accession and separation may be due to agency-level changes that affect employees’ job satisfaction or, in the case of accessions, expected job satisfaction. For example, scholars of turnover in the public sector often discuss person-organization fit (e.g., alignment of an individual’s values with the organization’s values). Changes in presidential administrations bring a new set of political appointees and, for some agencies, significant changes to policy goals, enforcement priorities, and, in general, how agencies pursue their missions. Employees who do not support these changes suffer reduction in person-organization fit and a loss of job satisfaction, making them more likely to exit the agency [e.g., 15]. However, individuals’ decisions to leave their agency may also be driven by idiosyncratic personal situations such as reaching retirement-age. Similarly, an individual’s decision to seek federal employment may be driven by a desire to support a presidential administration or relocating due to a spouse’s new job. Our goal is to use rates of separation and accession to make inferences about the effect of agency-level management choices on civil servants’ career decisions across the executive branch during the Trump administration.

Making inferences about agency-level management choices that affect employees’ career decisions directly using raw rates is problematic, as the following example illustrates. Suppose an agency with ten employees loses two people over some period. It will have a separation rate of 20 per 100, or more informally we might say a separation rate of 20%. If an agency staffed by a thousand loses one hundred employees over the same period, it will have a separation rate of 10%. The small agency’s raw separation rate is higher than the larger agency’s. Yet, it is not unlikely that one of the exiting employees at the smaller agency left for idiosyncratic reasons unrelated to management decisions at the agency. At the start of the Trump administration, federal agencies ranged in size from 2 non-seasonal full-time permanent employees at the Council of Economic Advisers to 680,237 at the Department of Defense. A single idiosyncratic turnover decision may have a larger influence on raw rates of smaller agencies than larger ones. Consider the bureaus of the 15 executive departments. Of the bureaus in the top 10% of separation rates, 36% have fewer than 100 employees. Similarly, of the bureaus in the bottom 10% of separation rates, 70% have fewer than 100 employees. However, only 22% of all bureaus have fewer than 100 employees. (We include only bureaus that exist in all 16 quarters of the Trump administration). Small bureaus are thus over-represented among bureaus with both very low and very high separation rates. Such a statistical artifact is unsurprising given their small size. Even so, it makes naive comparison of percentage changes in employment problematic.

Our approach to addressing this small-area issue relies on Bayesian hierarchical modeling. Our model formalizes intuitions and assumptions rooted in theories of political science and public administration. For ease of exposition, we will in this section refer primarily to separation rates, but conceptually the approach applies equally to accession rates. We assume that the process generating observed separation counts *S*_*i*_ in agency *i* is driven by (a) idiosyncratic career decisions related to a civil servants’ personal situations (e.g., retirement due to age, geographic relocation of a spouse’s job, birth of a child) and by (b) environmental conditions created by Presidents and their appointees. Because of such environmental pressures, each agency is assumed to have a particular separation rate parameter *μ*_*i*_. The actual number of separations an agency experiences over some time-frame *T* represents a draw from a probability distribution that depends on (i) the agency’s rate *μ*_*i*_, (ii) the number of employees an agency has *N*_*i*_, and (iii) the duration *T* that employment figures are observed across agencies. An agency’s separation rate parameter is related to the broader political environment of the executive branch as well as idiosyncratic factors particular to the agency. Therefore, the separation rate is *itself* a draw from some distribution that depends on latent, government-wide factors that are (by assumption) roughly constant throughout the Trump administration.

The following hierarchical model formalizes these notions:

$$\begin{array}{cc} S_{i} & ∼\text{Poisson}(T\cdot N_{i}\cdot \mu_{i})\\ \mu_{i} & ∼\text{Gamma}(\alpha ,\beta )\\ \alpha ,\beta & ∼\text{Cauchy}(0,2.5) \end{array}$$ (1)

To explicate our choices, we assume a Poisson distribution because *S*_*i*_ is count data. The Poisson distribution’s rate is the product of *T*, *N*_*i*_, and *μ*_*i*_, the former variables being observed constants and only *μ*_*i*_ being a statistical parameter. (We adapted this example and the model below from Gelman et al. [16], which discusses the problem of estimating cancer rates at the county level). *μ*_*i*_ is the primary quantity of interest. It is interpretable as an agency’s annual per capita rate of separations. In discussion, we will tend to focus on 100*μ_*i*_, which is the annual separation rate per 100 employees. The prior on *μ*_*i*_ is assumed to come from a Gamma distribution with parameters *α* and *β*, although in principle any positive continuous distribution might have been used. The Gamma’s parameters *α* and *β* arise independently from diffuse Cauchy priors. The scale parameter of the Cauchy is set at 2.5, but our estimates are not particularly sensitive to this choice.

To be clear, scholars and analysts who are interested in understanding changes in the size of the federal workforce across the various agencies and bureaus may prefer to use raw rates and counts because the adjusted rates will not equal the number of people that exited or joined an agency in a given time period. However, scholars and analysts interested in using employment records as a proxy for the environmental pressures acting on agencies will find that our estimates are better suited to their purposes.

Lastly, a key choice in this modeling approach is which agencies are drawn from a common distribution. We have assumed there exists a common distribution for all federal agencies for our agency-level analysis. (The estimated rates of EOP agencies may be especially sensitive to this assumption because they tend to be small. Therefore, in our analysis they will be pulled rather far from their raw rate and near the common rate for all agencies). We have assumed there exists a common distribution for each executive department for our bureau-level analysis. Bayesian methods similar to ours could be adapted to other assumptions about the appropriate grouping of agencies and bureaus. For example, agencies could be grouped by policy domain or their location in the executive establishment (e.g., EOP agencies, independent regulatory commissions) to reflect different assumptions about which agencies share a common environment.

### Exploring Bayesian adjustments

**Fig 1:**
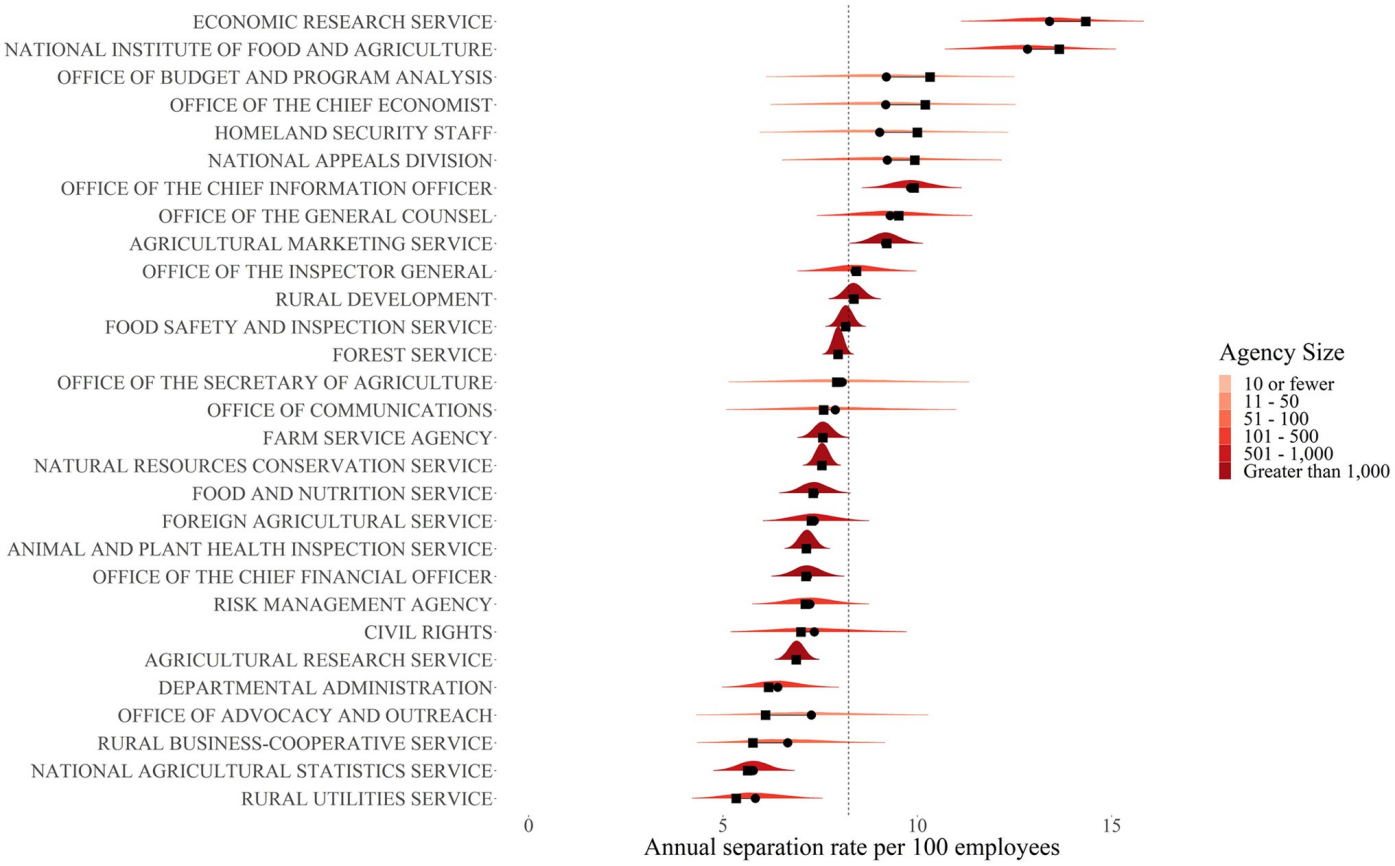
Adjusted vs. unadjusted separation rates at USDA with posterior distributions. Circles are the adjusted rate. Squares are the unadjusted rate. The dashed vertical line is the prior mean.

Our initial exploratory analysis begins with an investigation into how the Bayesian adjusted separation (accession) rates differ from the raw rates. Fig 1 presents a ridgeline plot of the posterior distributions of the separation rate *μ*_*i*_ for bureaus within the Department of Agriculture (USDA). Raw rates and posterior means are indicated to illustrate the size of the Bayesian adjustment. As expected, for small bureaus the adjustment is substantial, while for large bureaus the adjustment is negligible. The Office of the Budget and Program Analysis (OBPA) had 46 members prior to the start of the Trump administration and had 19 separations over the 4 years of our data. Its raw annual rate is 10.3 separations per 100 employees. A 90% credible interval on the bureau’s adjusted separation rate per 100 employees ranges from as low as 6.83 to as high as 11.9, with an expected rate of 9.21. Substantively, this credible interval is very wide. It goes from below the USDA’s average annual separation rate (indicated by the vertical dashed line) to as much as 144% of the USDA’s average. The observed rate is at the tail-end of a 90% credible interval. Indeed, the probability of observing a rate as large or bigger under the model is only 0.08. One could argue that the small Bayesian p-value may indicate that OBPA is truly different from the others, so that the model-based adjustment for OBPA is inappropriate. However, one should bear in mind that a small number of agencies outside a 90% credible interval is expected. There are only 3 bureaus out of 29 bureaus within USDA whose raw rate is outside the 90% credible interval for that agency’s *μ*_*i*_. Importantly, one of those bureaus—the Economic Research Service—was geographically relocated during the Trump administration causing a very high separation rate, as discussed below. Additionally, the Bayesian adjusted rates for Economic Research Service remains similar to the raw rate because of its relatively large size. For small reporting units like OBPA, one benefit of the model is that it reveals the sensitivity of the raw rates to small changes in individual employment decisions. If just a handful of people had stayed at OBPA, its raw separation rate would change drastically. The model-based adjustments are also useful in so far as it makes it harder for small agencies to drive aggregate patterns by pushing them toward the overall mean. The way the model handles larger agencies, such as the Agricultural Marketing Service (AMS), is quite different. The AMS had 1,943 employees prior to the start of the Trump administration and lost 716 over 4 years. Its raw annual separation rate of 9.21 differs little from its adjusted rate of 9.18, with a 90% credible interval ranging from 8.6 to 9.8. These rates were close to the average of all bureaus in USDA, however the fact that the AMS was “typical” is not the major explanation for its small adjustment. A brief inspection of Fig 1 will reveal that both the size of the common rate adjustment and the amount of uncertainty about the rate are largely determined by the bureau’s size.

**Fig 2:**
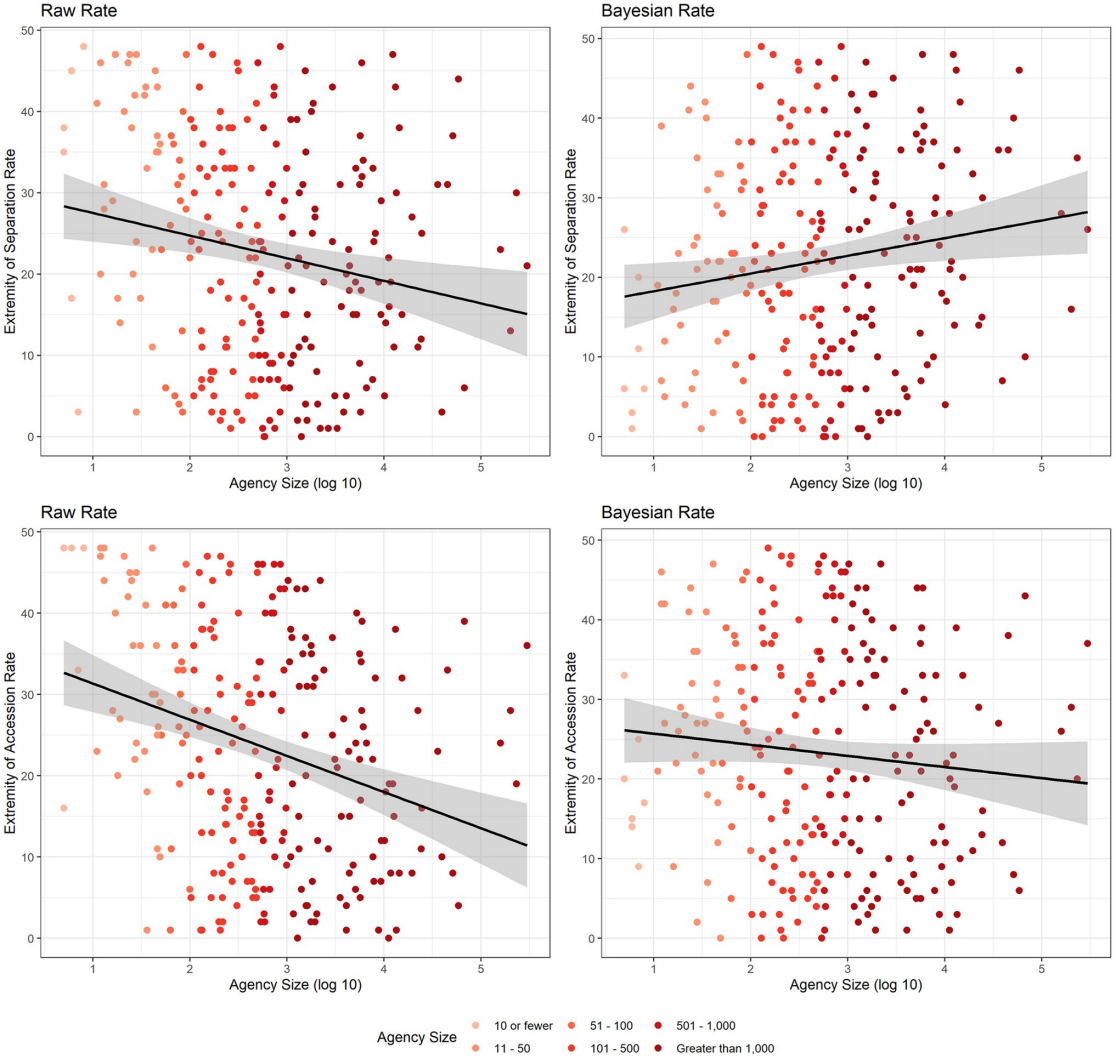
Relationship between bureau size and extremity of rates. Extremity is calculated by subtracting 50 from the percentage of agencies that have a rate less than or equal to each bureau’s rate and taking the absolute value. This transformation assigns the median rate a value of 0 and the minimum and maximum rates a value of 50. Each plot includes a fitted line from an OLS regression. The shaded regions around the line give the 95% confidence interval.

Fig 2 illustrates how the use of model-adjusted annual separation and accession rates changes the relationship between these rates and bureau size. The y-axis gives a measure of the extremity of the separation or accession rate for bureaus in every executive department. Specifically, the y-axis is: |pct. rank of the rate−50|. The measure assigns the median rate a value of 0 with assigned values approaching 50 as the percentile rank approaches 0 or 100.

As the scatter plots on the left show, there is a pronounced size trend in the raw rates. The clusters of bureaus near 50 on the y-axes show that small bureaus experienced both relatively greater raw rates of accessions and separations and relatively lesser raw rates of separations and accessions under Trump. In other words, small bureaus are disproportionately likely to have either very low or very high rates, which we regard as a statistical artifact. The right panels show the Bayesian adjusted rates. The cluster of small bureaus near 50 has been eliminated in both figures. While there remains a significant relationship between size and these rates in both cases, the magnitudes of the slope coefficients decline by 19% for separations and 69% for accessions. In terms of separations, the trend actually reverses. Analysis of the Bayesian rates suggests that larger bureaus on average faced higher separation and lower hiring rates than smaller bureaus.

## Employment trends during the Trump administration

**Fig 3:**
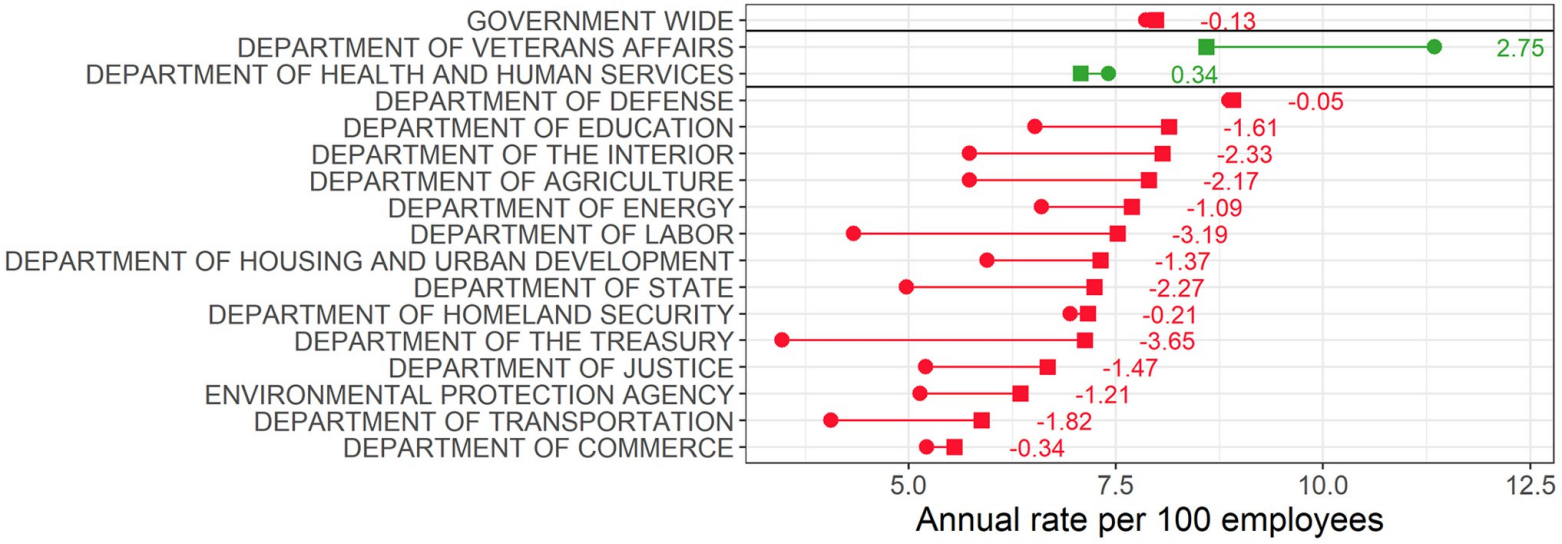
Accessions and separation during the Trump administration. ircles are accession rates. Squares are separation rates. The government-wide rates are raw rates.

We now put the Bayesian adjusted separation and accession rates to use in characterizing employment trends during the the Trump administration among permanent career civil servants. As a reminder, we subset the OPM data to non-seasonal full-time permanent civil servants, excluding non-career members of the Senior Executive Service, Schedule C appointees, and employees on the Executive Schedule pay plan. We also exclude certain small independent agencies listed in Table S.3 in the S1 File. Fig 3 shows the accession and separation rates for the government overall, the 15 executive departments, and the Environmental Protection Agency. To calculate the government-wide accession and separation rates, we summed accessions and separations for the employees and agencies in our data set and divided by total employees. Accession rates are circles and separation rates are squares. The (green) agencies above the horizontal line and below the government-wide rate gained employees (i.e., the accession rate exceeded the separation rate) while the 14 (red) agencies below the line lost employees. The numbers on the right give the accession rate less the separation rate which we will refer to as the net rate.

Fig 3 provides three key insights about employment trends at these agencies during the period under study. First, while government employment was nearly stable at an annualized rate of -0.13 per 100 employees, most departments lost employees. Second, the stability masks significant variation among agencies. The Department of Veterans Affairs grew significantly while, similar to the government overall, the net rates at the Departments of Defense, Health and Human Services, Homeland Security, and Commerce were near zero. The VA and Department of Defense account for 18% and 36% of total employment in our data, respectively, meaning that the large positive net rate at the VA and small negative rate at the DOD will be significant drivers of the government-wide rate. While the press has heavily covered employee separations at agencies like the State Department (net rate of -2.27) and the EPA (net rate of -1.21), many agencies have similar or larger net loss rates. (It is important to note that the State Department does not report data on Foreign Service Personnel, therefore, its net loss rate is likely understated given press reports about turnover among these personnel). In particular, the Departments of the Treasury, the Interior, and Labor have higher net loss rates and have gotten much less attention. Third, it is necessary to consider both separations and accessions to fully understand employment trends. For example, the Treasury Department has a middling separation rate but the lowest accession rate resulting in the largest net loss rate (-3.65).

**Fig 4:**
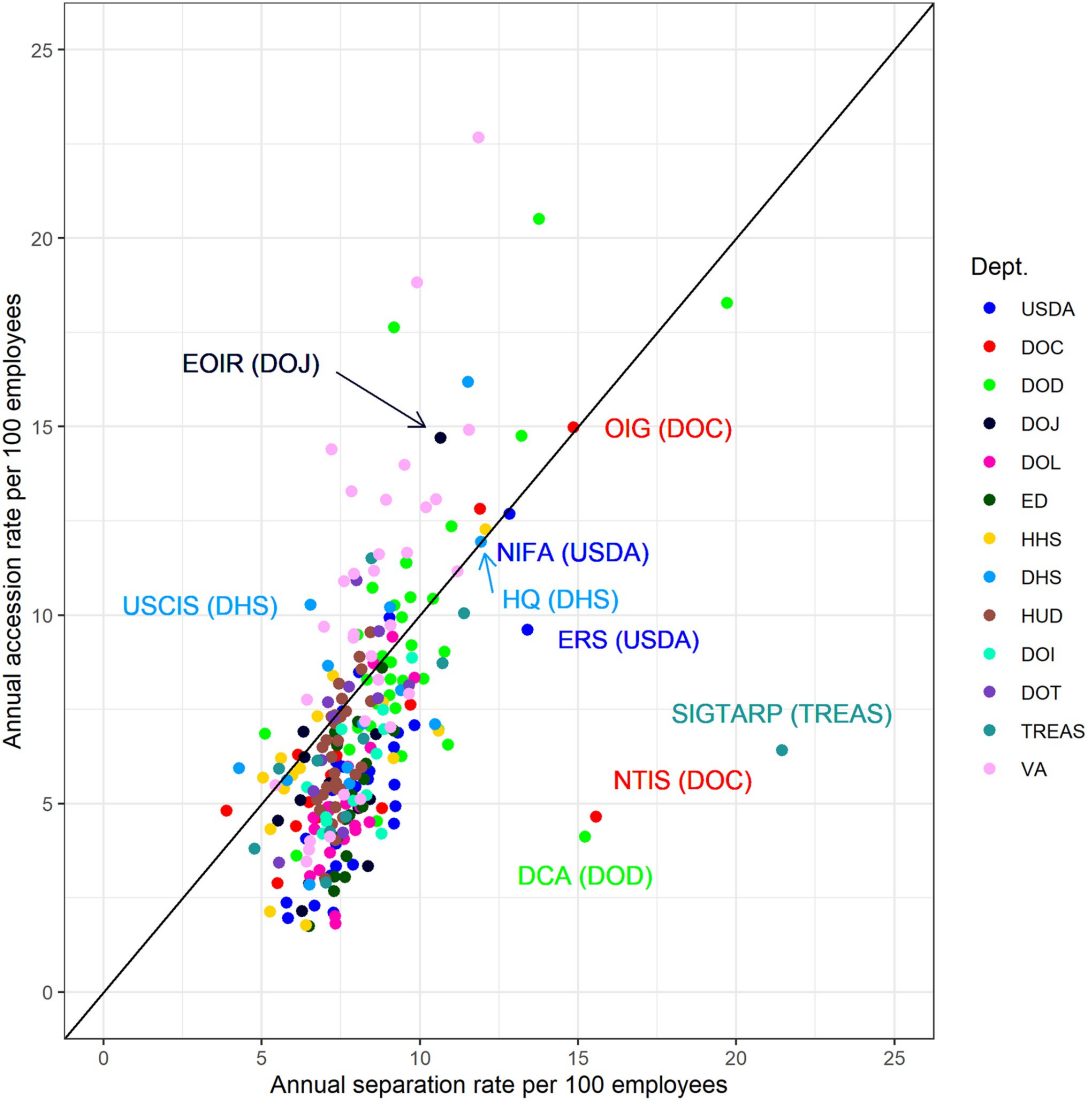
Separations and accessions within executive departments.

Fig 4 shows the joint distribution of separation and accession rates for bureaus within the 13 executive departments that provide data on their bureaus. The separation rate is on the x-axis, accession rate is on the y-axis, and the plot includes a 45-degree line. Observations below the line are agencies with a net loss (i.e., the separation rate exceeded the accession rate), observations above the line are cases with a net gain, and observations on (or near) the line are cases with equal (or near equal) separation and accession rates. As expected from the department-level analysis above, most of the bureaus with a net gain are in the Department of Veterans Affairs (VA, pink dots), the Department of Defense (DOD, green dots), or the Department of Homeland Security (DHS, light blue dots).

The Economic Research Service (ERS) and National Institute of Food and Agriculture (NIFA) within the Department of Agriculture are two bureaus one would expect to be deep in the lower right portion of Fig 4. The Trump administration relocated these scientific bureaus from Washington, DC, to Kansas City, MO, in October 2019. The employees of these agencies generally opposed the move and many chose to leave the bureaus rather than relocate [17] resulting in large separation rates. Our analysis shows that each bureau was largely able to replace the staff who left. The separation outliers—Office of the Inspector General and the National Technical Information Service in Commerce, the Special Inspector General for the Troubled Asset Relief Program (SIGTARP) in Treasury, and the Defense Commissary Agency in DOD—are not clearly related to priorities of the Trump administration.

One priority of the Trump administration that did have clear effects on personnel is immigration. The United States Citizenship and Immigration Services (USCIS), the agency in Homeland Security responsible for managing lawful immigration, experienced large net growth. Similarly, the Executive Office for Immigration Review, the agency in the Department of Justice responsible for adjudicating immigration cases, also experienced large net growth. Both of these agencies would have required more employees to enforce the Trump administration’s immigration policies. These bureaus standout relative to other bureaus within the same department.

A final observation from Fig 4 is that we should focus our attention on bureaus with high rates of separations and accessions that are near equal in addition to bureaus with high net positive or net negative rates. For example, DHS Headquarters sits on the 45-degree line with a net rate of 0. However, its rates of accessions and separations were 12 per hundred employees per year, which is likely due to frequent leadership changes at DHS during the Trump administration. We see similar large but nearly equal rates of accessions and separations in NIFA in USDA due to the bureau’s geographic relocation noted above. Bureaus with high but near equal rates of separations and accessions will have a stable number of total employees but this stability belies a churn in employees that may reduce capacity as employees learn their new roles and subordinates adjust to new leadership with varying management styles and priorities. A high rate of churn is likely to be especially problematic when it occurs among senior leadership.

**Fig 5:**
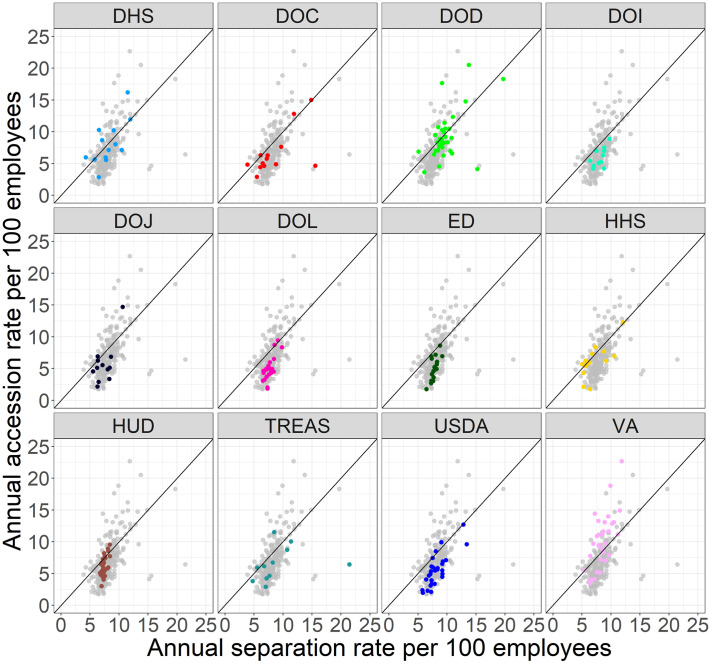
Separations and accessions by department. We omit DOT due to space constraints because it’s bureaus are clustered near the 45-degree line. See Fig. S.1 in the SI for this plot inlcuding DOT.

It is difficult to see variation in Fig 4 among the large mass of points in the lower left quadrant. To elucidate variation in this portion of the plot, Fig 5 contains a separate plot for each executive department, highlighting the points that correspond to bureaus within that department. Fig 5 shows that departments can generally be put into three categories:

1. Departments with a net positive growth rate or near break-even net rate that contain bureaus with heterogeneous growth rates—VA, HHS, DHS, and DOD;
2. Departments with a net negative growth rate that contain bureaus with heterogeneous growth rates—DOC, DOT, and HUD.
3. Departments with a net negative growth rate that contain bureaus with mostly negative growth rates—DOI, DOJ, DOL, ED, TREAS, and USDA.

When interpreting Figs 3 and 5, it is important to note that Fig 5 does not indicate bureau size which affects the influence of the bureau rates in Fig 5 on the department rates in Fig 3. For example, Treasury has a net department rate of -3.65 which largely reflects the employment trends at the Internal Revenue service (separation rate of 7.04, accession rate of 2.91, and a net rate of -4.13) because the IRS comprises 82% of Treasury’s 81,948 employees while SIGTARP (an outlier bureau in Fig 4) comprises 0.16%.

## Trump administration in historical context

**Fig 6:**
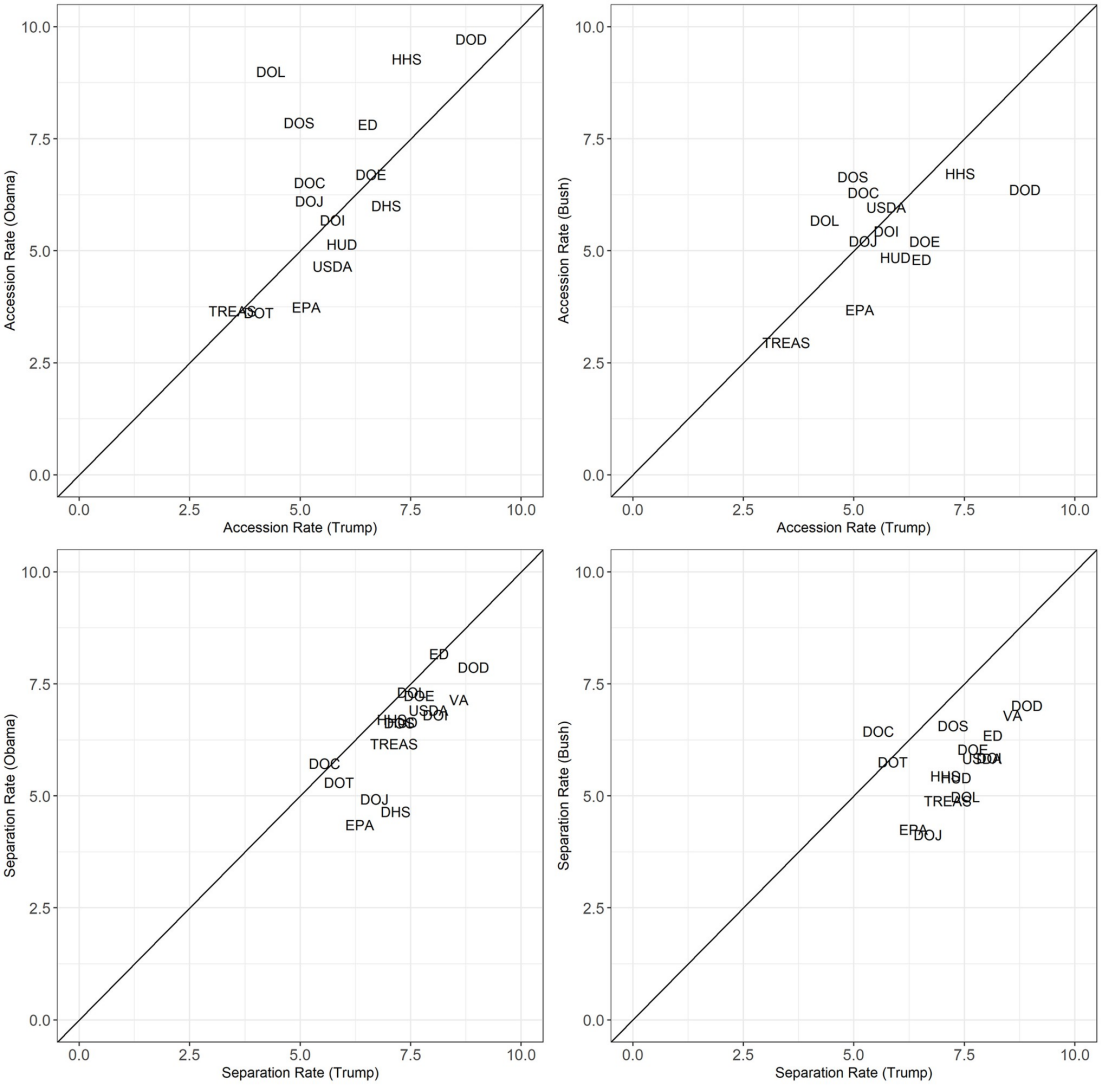
Bayesian adjusted accession and separation rates across administrations. We omit the observation for DOT in the Bush administration because it is an outlier (an accession rate of 20.84) to better show variation among other agencies.

**Fig 7:**
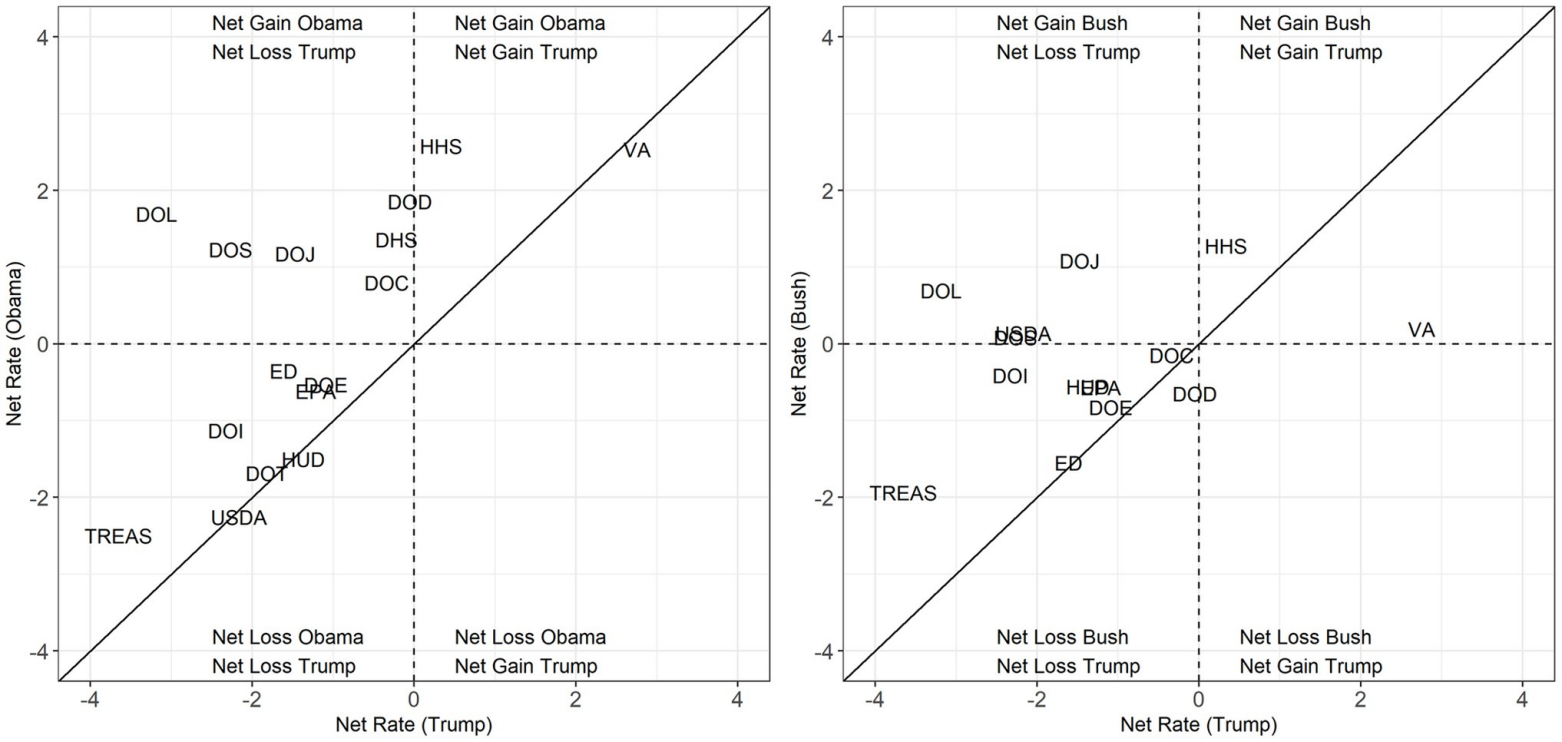
Bayesian adjusted net rates across administrations. We omit the observation for DOT in the Bush administration because it is an outlier (net accession rate of 15.08) to better show variation among other agencies.

Our analysis thus far characterizes employment trends in the Trump administration, but does not place the Trump administration in historical context. For example, are the accession and separation rates at the EPA in the Trump administration typical of Republican administrations? To answer questions like this, we applied our Bayesian hierarchical model to data for the first terms of Presidents George W. Bush and Barack Obama. Figs 6 and 7 compare accession and separation rates and net rates, respectively, between the Trump administration and the Bush and Obama administrations for the 15 executive departments and the EPA.

In Fig 6, the Trump administration is on the x-axes and the Bush and Obama administrations are on the y-axes and each plot includes a 45-degree line. Agencies above (below) the 45-degree line have a higher (lower) accession or separation rate in the Bush or Obama administration than the Trump administration and agencies near the line have nearly equivalent rates. While no clear pattern is evident when comparing accession rates, the lower two panels in Fig 6 show that separation rates in the Trump administration were higher than in either of the previous administrations, especially the Bush administration. Turning to the net rates in Fig 7, we see that the larger separation rates in the Trump administration result in net rates that are equivalent or larger (indicating either a smaller net loss or net growth rather than net loss) in the Bush and Obama administrations. It is also important to note that a small difference in annual rates per 100 employees can result in significant differences when both agency size and a full four-year term are considered. For example, the net rate at the EPA was -1.21 under Trump and -0.62 under Obama for a difference of -0.59. However, those net rates correspond to a net loss of 696 employees (4.84% of total employees at the start of the administration) under Trump compared to 402.52 (4%) under Obama.

Three departments—Justice, Labor, and State—experienced a significant reversal in trend during the Trump administration. Fig 7 shows that these departments had some of the largest net growth rates during the Bush and Obama administrations and some of the largest net loss rates during Trump administration.

## Discussion

Numerous agencies have experienced significant staff reductions during the course of the Trump administration. Members of Congress, the media, and other watchdogs have previously identified some of these agencies as in crisis, notably the State Department and Environmental Protection Agency. Our analysis shows that the loss of employees at the State Department and Environmental Protection Agency are not outliers. Other agencies have suffered significant loss of staff according to our analysis—in particular the Departments of the Interior, Labor, and the Treasury—but have received much less attention.

The relocation of the Economic Research Service and the National Institute of Food and Agriculture also received significant attention from the press and Congress [17]. Our analysis shows ERS and NIFA are clear outliers among executive department bureaus. The Congressional Research Service reports that 75% of employees affected by the relocation left the agencies rather than relocate [17]. Our analysis shows that these agencies were largely able to replace these staff, but we may nonetheless worry about reduced agency capacity as new staff learn their jobs and staff adjust to new leadership.

Our analysis also identified agencies that have been subject to intense political conflict which has had minimal effect on staffing. For example, the Federal Bureau of Investigation had a net rate of -0.98 with separation and accession rates of about 6. Similarly, Immigration and Customs Enforcement had a net rate of -0.18 also with separation and accession rates of about 6. The lack of growth at ICE despite efforts by the Trump administration that began in 2017 to hire 10,000 additional agents suggest that ICE’s stability may indicate that ICE is a bureau without sufficient staff to accomplish its mission during the Trump administration. Customs and Border Protection, which was paired with ICE in the hiring push for agents, had a net rate of 1.66. Had the hiring push at ICE been successful, the agency would have grown by about 50% (equivalent to a net rate of 50), which may have been unrealistic given the hiring process at ICE. (It takes more than 200 days to hire an ICE agent. The hiring process includes an entrance exam, physical exam, medical exam, drug test, and background investigation. After hiring, there’s a 16-week basic training program, which includes a five-week Spanish language course [18]). We would have expected growth at ICE to be similar to growth at CBP and USCIS in DHS or EOIR in DOJ.

The Equal Employment Opportunity Commission (EEOC) and the Minority Business Development Agency (MBDA), which work to further civil rights, also exhibited stress due to personnel changes. The EEOC (separation rate of 9.41, accession rate of 7.01, net rate of -2.39) had accession and separation rates near the 60th percentile for all agencies despite its small net loss rate, indicating a high rate of churn. The MBDA in DOC (separation rate of 8.82, accession rate of 4.89, net rate of -3.93), the only federal agency solely dedicated to the growth and global competitiveness of minority business enterprises per its website, had a large net loss rate due to a separation rate at the 78th percentile and an accession rate at the 28th percentile among all bureaus. Similarly, the Offices, Boards and Divisions reporting unit in DOJ (separation rate of 8.35, accession rate of 3.34, net rate of -5.02), which includes the Civil Rights Division, had a separation rate at the 71th percentile and an accession rate at the 11th percentile among all bureaus.

Finally, we compared employment trends in the executive departments and EPA in the Trump administration to the Obama and Bush administrations. The primary difference between the Trump administration and the prior two administrations is the elevated separation rates under Trump at nearly all of these agencies, leaving relative net rates in the Trump administration compared to the other two to be determined by whether the agency had a similarly elevated accession rate.

Many members of Congress, journalists, and scholars expressed concern about turnover in the federal workforce during the Trump administration. Our analysis helps such individuals understand employment trends across the vast executive branch and places them in recent historical context. While the federal workforce ended the Trump administration essentially where it began, that observation belies significant heterogeneity across agencies and bureaus. Some of these concerned observers focused attention on the State Department and EPA, but our analysis shows that the net loss rates at these agencies were not uncommon. Agencies like the Department of Interior and bureaus like the Minority Business Development Agency experienced larger net loss rates and have received much less attention. We also highlighted that a net rate near zero may mask large, near equal separation and accessions rates indicative of a high rate of employee churn that may harm performance. Simply looking at net changes in employee counts will not identify these cases.

## Conclusion

The personnel policies of the Trump administration have focused public attention on staffing at federal agencies. We explained that using raw counts to calculate separation and accession rates at federal agencies to make inferences about the effect of these personnel policies on working conditions is problematic due to significant heterogeneity in agency size. We presented a method to estimate rates that can be used to make reasonable interagency comparisons. Similar methods can be applied to make comparisons of other types of rates among agencies of varying sizes, including using survey data (e.g., the Federal Employee Viewpoint Survey) or measures of agency politicization based on counts of appointees in an agency. We then used these estimates to characterize the impact of the Trump administration’s policies on staffing at federal agencies. We are making available our Bayesian adjusted separation and accession rates for 80 agencies and 250 bureaus in the Trump administration as well as the corresponding rates in the Bush and Obama administrations.

Our study yields three primary conclusions. Total government employment at the end of the Trump presidency was approximately where it was when his presidency began. However, there was significant variation in growth rates across departments (and bureaus) with some growing, others stable, and many shrinking materially. Although this variation was in many cases not as strongly related to the policy priorities of the Trump Presidency as one might expect, we do note that the large growth rates in immigration-focused and veteran-focused agencies were consistent with these priorities. Further, signs of stress at offices focused on civil rights are also consistent with the Trump administration priorities, at least as articulated by administration critics. Finally, we show that relative to the first terms of the previous two presidents, separation rates in the Trump administration were markedly higher.

## Supporting information

S1 File (PDF)
