# Supplementary material for: Identifying bureaus with substantial personnel change during the Trump administration: A Bayesian approach
Source: PLoS One. 2023 Jan 18;18(1):e0278458. doi: 10.1371/journal.pone.0278458 (PMC9847970; doi:10.1371/journal.pone.0278458)
Supplement: S1 File — (PDF) [file pone.0278458.s001.pdf]

Supporting Information for “Identifying bureaus with subtantial personnel change during the Trump administration: A Bayesian approach”

S1 Discussion of OPM Data and Conding Decisions

We make several minor and technical adjustments to the OPM data. The OPM data codes the Departments of the Air Force, Army and Navy as separate departments from the Department of Defense. We recode the military service branches and treat them as a subcomponents of the Department of Defense, which they are. The Department of State does not report data on any subcomponents. The Department of Energy reports data on only one subcomponent, the Federal Energy Regulatory Commission. Section S2 below includes a list of small independent agencies (e.g., scholarship foundations) that we exclude from our analysis because we do not think they share a common political environment with other federal agencies, making the assumption that these agencies’ separation and accessions rates are drawn from a common distribution with other agencies inappropriate. See the Model and Assumptions section for further discussion. We also exclude the Peace Corps because it only has one permanent full-time employee.

See OPM’s Guide to Processing Personnel Actions, Chapter 21, p. 21-3 for the definition of mass transfer.

S2 Bayesian Adjusted Rate for agencies and bureaus.

Tables S1 and S2 provide the Bayesian adjusted rates for agencies and bureaus, respectively.

Table S.1. Bayesian Adjusted Rates in Agencies

Agency	Pres.	Sep. Rate	Acc. Rate	Net Rate
ADMINISTRATIVE CONFERENCE OF THE UNITED STATES	Trump	15.94	15.56	-0.38
AFRICAN DEVELOPMENT FOUNDATION	Trump	17.90	18.32	0.42
AFRICAN DEVELOPMENT FOUNDATION	Obama	11.83	16.77	4.93
AFRICAN DEVELOPMENT FOUNDATION	Bush	15.26	8.86	-6.39
CHEMICAL SAFETY AND HAZARD INVESTIGATION BOARD	Trump	12.79	9.90	-2.89
CHEMICAL SAFETY AND HAZARD INVESTIGATION BOARD	Obama	8.34	13.59	5.24
CHEMICAL SAFETY AND HAZARD INVESTIGATION BOARD	Bush	11.49	14.08	2.60
COMMISSION ON CIVIL RIGHTS	Trump	9.87	13.97	4.11
COMMISSION ON CIVIL RIGHTS	Obama	8.57	4.79	-3.78
COMMISSION ON CIVIL RIGHTS	Bush	11.44	6.83	-4.60
COMMODITY FUTURES TRADING COMMISSION	Trump	4.51	3.75	-0.76
COMMODITY FUTURES TRADING COMMISSION	Obama	8.64	19.57	10.93
COMMODITY FUTURES TRADING COMMISSION	Bush	7.35	5.73	-1.62
CONSUMER FINANCIAL PROTECTION BUREAU	Trump	7.22	5.50	-1.72
CONSUMER PRODUCT SAFETY COMMISSION	Trump	10.05	8.23	-1.82
CONSUMER PRODUCT SAFETY COMMISSION	Obama	8.55	13.32	4.78
CONSUMER PRODUCT SAFETY COMMISSION	Bush	7.70	6.63	-1.07
CORPORATION FOR NATIONAL AND COMMUNITY SERVICE	Trump	18.25	10.19	-8.06
CORPORATION FOR NATIONAL AND COMMUNITY SERVICE	Obama	10.95	12.89	1.94
CORPORATION FOR NATIONAL AND COMMUNITY SERVICE	Bush	8.51	4.50	-4.01
COUNCIL OF ECONOMIC ADVISERS	Trump	12.80	23.43	10.63
COUNCIL OF ECONOMIC ADVISERS	Obama	8.58	7.95	-0.63
COUNCIL OF ECONOMIC ADVISERS	Bush	31.74	32.76	1.02
COUNCIL ON ENVIRONMENTAL QUALITY/OFFICE OF ENVIRONMENTAL QUALITY	Trump	10.78	14.67	3.89
COUNCIL ON ENVIRONMENTAL QUALITY/OFFICE OF ENVIRONMENTAL QUALITY	Obama	8.21	7.15	-1.07

Table S.1. Bayesian Adjusted Rates in Agencies *(continued)*

Agency	Pres.	Sep. Rate	Acc. Rate	Net Rate
COUNCIL ON ENVIRONMENTAL QUALITY/OFFICE OF ENVIRONMENTAL QUALITY	Bush	13.13	16.25	3.12
DEFENSE NUCLEAR FACILITIES SAFETY BOARD	Trump	10.83	6.72	-4.11
DEFENSE NUCLEAR FACILITIES SAFETY BOARD	Obama	6.56	11.27	4.71
DEFENSE NUCLEAR FACILITIES SAFETY BOARD	Bush	6.33	6.68	0.35
DEPARTMENT OF AGRICULTURE	Trump	7.90	5.73	-2.17
DEPARTMENT OF AGRICULTURE	Obama	6.92	4.66	-2.26
DEPARTMENT OF AGRICULTURE	Bush	5.84	5.98	0.14
DEPARTMENT OF COMMERCE	Trump	5.55	5.22	-0.34
DEPARTMENT OF COMMERCE	Obama	5.72	6.52	0.80
DEPARTMENT OF COMMERCE	Bush	6.45	6.30	-0.15
DEPARTMENT OF DEFENSE	Trump	8.92	8.87	-0.05
DEPARTMENT OF DEFENSE	Obama	7.87	9.72	1.85
DEPARTMENT OF DEFENSE	Bush	7.02	6.37	-0.65
DEPARTMENT OF EDUCATION	Trump	8.14	6.53	-1.61
DEPARTMENT OF EDUCATION	Obama	8.17	7.82	-0.35
DEPARTMENT OF EDUCATION	Bush	6.35	4.80	-1.55
DEPARTMENT OF ENERGY	Trump	7.69	6.60	-1.09
DEPARTMENT OF ENERGY	Obama	7.24	6.71	-0.53
DEPARTMENT OF ENERGY	Bush	6.04	5.21	-0.83
DEPARTMENT OF HEALTH AND HUMAN SERVICES	Trump	7.07	7.41	0.34
DEPARTMENT OF HEALTH AND HUMAN SERVICES	Obama	6.71	9.28	2.57
DEPARTMENT OF HEALTH AND HUMAN SERVICES	Bush	5.45	6.73	1.28
DEPARTMENT OF HOMELAND SECURITY	Trump	7.16	6.95	-0.21
DEPARTMENT OF HOMELAND SECURITY	Obama	4.65	6.01	1.36
DEPARTMENT OF HOUSING AND URBAN DEVELOPMENT	Trump	7.31	5.95	-1.37

Table S.1. Bayesian Adjusted Rates in Agencies *(continued)*

Agency	Pres.	Sep. Rate	Acc. Rate	Net Rate
DEPARTMENT OF HOUSING AND URBAN DEVELOPMENT	Obama	6.64	5.14	-1.50
DEPARTMENT OF HOUSING AND URBAN DEVELOPMENT	Bush	5.41	4.85	-0.56
DEPARTMENT OF JUSTICE	Trump	6.68	5.21	-1.47
DEPARTMENT OF JUSTICE	Obama	4.93	6.11	1.18
DEPARTMENT OF JUSTICE	Bush	4.13	5.22	1.08
DEPARTMENT OF LABOR	Trump	7.52	4.34	-3.19
DEPARTMENT OF LABOR	Obama	7.31	9.00	1.69
DEPARTMENT OF LABOR	Bush	4.99	5.68	0.69
DEPARTMENT OF STATE	Trump	7.24	4.98	-2.27
DEPARTMENT OF STATE	Obama	6.63	7.86	1.23
DEPARTMENT OF STATE	Bush	6.57	6.65	0.08
DEPARTMENT OF THE INTERIOR	Trump	8.06	5.74	-2.33
DEPARTMENT OF THE INTERIOR	Obama	6.81	5.68	-1.13
DEPARTMENT OF THE INTERIOR	Bush	5.85	5.44	-0.41
DEPARTMENT OF THE TREASURY	Trump	7.13	3.47	-3.65
DEPARTMENT OF THE TREASURY	Obama	6.16	3.66	-2.50
DEPARTMENT OF THE TREASURY	Bush	4.89	2.96	-1.94
DEPARTMENT OF TRANSPORTATION	Trump	5.88	4.06	-1.82
DEPARTMENT OF TRANSPORTATION	Obama	5.30	3.62	-1.68
DEPARTMENT OF TRANSPORTATION	Bush	5.76	20.84	15.08
DEPARTMENT OF VETERANS AFFAIRS	Trump	8.59	11.35	2.75
DEPARTMENT OF VETERANS AFFAIRS	Obama	7.15	9.69	2.54
DEPARTMENT OF VETERANS AFFAIRS	Bush	6.79	6.99	0.19
ELECTION ASSISTANCE COMMISSION	Trump	18.78	58.64	39.86
ELECTION ASSISTANCE COMMISSION	Obama	7.16	9.09	1.93
ENVIRONMENTAL PROTECTION AGENCY	Trump	6.35	5.14	-1.21

Table S.1. Bayesian Adjusted Rates in Agencies *(continued)*

Agency	Pres.	Sep. Rate	Acc. Rate	Net Rate
ENVIRONMENTAL PROTECTION AGENCY	Obama	4.36	3.74	-0.62
ENVIRONMENTAL PROTECTION AGENCY	Bush	4.25	3.69	-0.57
EQUAL EMPLOYMENT OPPORTUNITY COMMISSION	Trump	9.41	7.01	-2.39
EQUAL EMPLOYMENT OPPORTUNITY COMMISSION	Obama	7.46	8.61	1.15
EQUAL EMPLOYMENT OPPORTUNITY COMMISSION	Bush	6.22	3.65	-2.57
EXPORT-IMPORT BANK OF THE UNITED STATES	Trump	10.44	6.76	-3.68
EXPORT-IMPORT BANK OF THE UNITED STATES	Obama	8.08	10.83	2.75
EXPORT-IMPORT BANK OF THE UNITED STATES	Bush	8.15	8.52	0.37
FARM CREDIT ADMINISTRATION	Trump	10.70	11.09	0.39
FARM CREDIT ADMINISTRATION	Obama	7.78	8.25	0.46
FARM CREDIT ADMINISTRATION	Bush	5.17	3.68	-1.49
FARM CREDIT SYSTEM INSURANCE CORPORATION	Trump	7.61	6.20	-1.41
FARM CREDIT SYSTEM INSURANCE CORPORATION	Obama	6.53	8.04	1.51
FARM CREDIT SYSTEM INSURANCE CORPORATION	Bush	9.11	7.89	-1.22
FEDERAL COMMUNICATIONS COMMISSION	Trump	6.89	4.93	-1.96
FEDERAL COMMUNICATIONS COMMISSION	Obama	5.02	3.65	-1.37
FEDERAL COMMUNICATIONS COMMISSION	Bush	4.90	5.09	0.18
FEDERAL DEPOSIT INSURANCE CORPORATION	Trump	6.79	5.86	-0.93
FEDERAL DEPOSIT INSURANCE CORPORATION	Obama	5.60	9.13	3.53
FEDERAL DEPOSIT INSURANCE CORPORATION	Bush	6.95	2.12	-4.83
FEDERAL ELECTION COMMISSION	Trump	7.52	6.34	-1.18
FEDERAL ELECTION COMMISSION	Obama	7.35	6.22	-1.13
FEDERAL ELECTION COMMISSION	Bush	9.33	11.61	2.29
FEDERAL EMERGENCY MANAGEMENT AGENCY	Bush	3.58	3.17	-0.41
FEDERAL FINANCIAL INSTITUTIONS EXAMINATION COUNCIL	Trump	6.06	4.87	-1.19
FEDERAL FINANCIAL INSTITUTIONS EXAMINATION COUNCIL	Obama	6.39	11.81	5.42
FEDERAL FINANCIAL INSTITUTIONS EXAMINATION COUNCIL	Bush	8.15	7.82	-0.32

Table S.1. Bayesian Adjusted Rates in Agencies *(continued)*

Agency	Pres.	Sep. Rate	Acc. Rate	Net Rate
FEDERAL HOUSING FINANCE AGENCY	Trump	6.19	7.43	1.24
FEDERAL HOUSING FINANCE BOARD	Obama	2.86	3.43	0.57
FEDERAL HOUSING FINANCE BOARD	Bush	14.61	15.52	0.91
FEDERAL LABOR RELATIONS AUTHORITY	Trump	12.18	7.07	-5.11
FEDERAL LABOR RELATIONS AUTHORITY	Obama	7.25	10.54	3.28
FEDERAL LABOR RELATIONS AUTHORITY	Bush	10.60	7.17	-3.43
FEDERAL MARITIME COMMISSION	Trump	10.95	9.47	-1.49
FEDERAL MARITIME COMMISSION	Obama	6.15	7.55	1.40
FEDERAL MARITIME COMMISSION	Bush	8.17	9.61	1.44
FEDERAL MEDIATION AND CONCILIATION SERVICE	Trump	9.46	8.07	-1.39
FEDERAL MEDIATION AND CONCILIATION SERVICE	Obama	6.45	5.72	-0.74
FEDERAL MEDIATION AND CONCILIATION SERVICE	Bush	6.49	5.22	-1.27
FEDERAL MINE SAFETY AND HEALTH REVIEW COMMISSION	Trump	9.21	10.59	1.39
FEDERAL MINE SAFETY AND HEALTH REVIEW COMMISSION	Obama	8.57	11.70	3.13
FEDERAL MINE SAFETY AND HEALTH REVIEW COMMISSION	Bush	9.75	6.55	-3.20
FEDERAL RETIREMENT THRIFT INVESTMENT BOARD	Trump	10.34	13.71	3.38
FEDERAL RETIREMENT THRIFT INVESTMENT BOARD	Obama	8.87	25.18	16.32
FEDERAL RETIREMENT THRIFT INVESTMENT BOARD	Bush	7.14	6.76	-0.37
FEDERAL TRADE COMMISSION	Trump	7.74	7.23	-0.51
FEDERAL TRADE COMMISSION	Obama	8.22	8.30	0.08
FEDERAL TRADE COMMISSION	Bush	8.07	7.29	-0.78
GENERAL SERVICES ADMINISTRATION	Trump	6.63	6.22	-0.42
GENERAL SERVICES ADMINISTRATION	Obama	6.30	7.22	0.92
GENERAL SERVICES ADMINISTRATION	Bush	6.42	5.74	-0.68
GOVERNMENT PRINTING OFFICE	Trump	6.34	4.43	-1.91
GOVERNMENT PRINTING OFFICE	Obama	7.92	2.63	-5.29
GOVERNMENT PRINTING OFFICE	Bush	10.40	4.13	-6.27

Table S.1. Bayesian Adjusted Rates in Agencies *(continued)*

Agency	Pres.	Sep. Rate	Acc. Rate	Net Rate
INTER-AMERICAN FOUNDATION	Trump	9.95	11.91	1.95
INTER-AMERICAN FOUNDATION	Obama	9.73	9.22	-0.51
INTER-AMERICAN FOUNDATION	Bush	14.28	15.14	0.86
MEDICARE PAYMENT ADVISORY COMMISSION	Trump	10.94	12.16	1.22
MEDICARE PAYMENT ADVISORY COMMISSION	Obama	8.74	13.75	5.01
MEDICARE PAYMENT ADVISORY COMMISSION	Bush	14.17	15.86	1.68
MERIT SYSTEMS PROTECTION BOARD	Trump	9.96	8.78	-1.18
MERIT SYSTEMS PROTECTION BOARD	Obama	6.64	5.11	-1.54
MERIT SYSTEMS PROTECTION BOARD	Bush	7.96	8.78	0.82
MILLENNIUM CHALLENGE CORPORATION	Trump	9.93	12.26	2.34
MILLENNIUM CHALLENGE CORPORATION	Obama	13.74	14.60	0.87
NATIONAL AERONAUTICS AND SPACE ADMINISTRATION	Trump	5.62	5.93	0.31
NATIONAL AERONAUTICS AND SPACE ADMINISTRATION	Obama	4.67	3.24	-1.43
NATIONAL AERONAUTICS AND SPACE ADMINISTRATION	Bush	4.33	3.77	-0.56
NATIONAL ARCHIVES AND RECORDS ADMINISTRATION	Trump	9.70	6.54	-3.15
NATIONAL ARCHIVES AND RECORDS ADMINISTRATION	Obama	6.41	6.34	-0.07
NATIONAL ARCHIVES AND RECORDS ADMINISTRATION	Bush	9.05	10.53	1.49
NATIONAL CREDIT UNION ADMINISTRATION	Trump	6.93	5.13	-1.80
NATIONAL CREDIT UNION ADMINISTRATION	Obama	8.66	15.54	6.87
NATIONAL CREDIT UNION ADMINISTRATION	Bush	5.30	3.05	-2.25
NATIONAL FOUNDATION ON THE ARTS AND THE HUMANITIES	Trump	9.92	9.55	-0.38
NATIONAL FOUNDATION ON THE ARTS AND THE HUMANITIES	Obama	9.37	7.23	-2.14
NATIONAL FOUNDATION ON THE ARTS AND THE HUMANITIES	Bush	6.70	7.20	0.50
NATIONAL LABOR RELATIONS BOARD	Trump	7.21	2.31	-4.91
NATIONAL LABOR RELATIONS BOARD	Obama	7.30	6.38	-0.92
NATIONAL LABOR RELATIONS BOARD	Bush	7.77	5.81	-1.96

Table S.1. Bayesian Adjusted Rates in Agencies *(continued)*

Agency	Pres.	Sep. Rate	Acc. Rate	Net Rate
NATIONAL SCIENCE FOUNDATION	Trump	7.43	6.66	-0.77
NATIONAL SCIENCE FOUNDATION	Obama	7.03	6.57	-0.45
NATIONAL SCIENCE FOUNDATION	Bush	7.60	9.27	1.67
NATIONAL SECURITY COUNCIL	Trump	8.68	5.12	-3.56
NATIONAL SECURITY COUNCIL	Obama	6.89	4.54	-2.35
NATIONAL SECURITY COUNCIL	Bush	23.60	28.83	5.24
NATIONAL TRANSPORTATION SAFETY BOARD	Trump	6.42	5.41	-1.01
NATIONAL TRANSPORTATION SAFETY BOARD	Obama	6.32	7.22	0.90
NATIONAL TRANSPORTATION SAFETY BOARD	Bush	7.85	6.35	-1.50
NUCLEAR REGULATORY COMMISSION	Trump	6.09	1.74	-4.35
NUCLEAR REGULATORY COMMISSION	Obama	5.10	3.40	-1.71
NUCLEAR REGULATORY COMMISSION	Bush	3.53	8.07	4.54
OCCUPATIONAL SAFETY AND HEALTH REVIEW COMMISSION	Trump	9.10	7.63	-1.46
OCCUPATIONAL SAFETY AND HEALTH REVIEW COMMISSION	Obama	8.82	11.23	2.41
OCCUPATIONAL SAFETY AND HEALTH REVIEW COMMISSION	Bush	10.04	5.40	-4.64
OFC OF NAVAJO AND HOPI INDIAN RELOCATION	Obama	6.86	4.54	-2.31
OFC OF NAVAJO AND HOPI INDIAN RELOCATION	Bush	4.46	0.96	-3.50
OFFICE OF ADMINISTRATION	Trump	16.36	15.73	-0.64
OFFICE OF ADMINISTRATION	Obama	11.51	11.97	0.46
OFFICE OF ADMINISTRATION	Bush	11.86	16.77	4.91
OFFICE OF GOVERNMENT ETHICS	Trump	8.55	9.42	0.87
OFFICE OF GOVERNMENT ETHICS	Obama	8.42	8.88	0.46
OFFICE OF GOVERNMENT ETHICS	Bush	12.43	13.06	0.64
OFFICE OF MANAGEMENT AND BUDGET	Trump	12.79	14.11	1.31
OFFICE OF MANAGEMENT AND BUDGET	Obama	9.16	7.99	-1.16
OFFICE OF MANAGEMENT AND BUDGET	Bush	9.60	10.21	0.61
OFFICE OF NATIONAL DRUG CONTROL POLICY	Trump	8.88	2.45	-6.43

Table S.1. Bayesian Adjusted Rates in Agencies *(continued)*

Agency	Pres.	Sep. Rate	Acc. Rate	Net Rate
OFFICE OF NATIONAL DRUG CONTROL POLICY	Obama	8.43	8.56	0.13
OFFICE OF NATIONAL DRUG CONTROL POLICY	Bush	8.95	7.55	-1.40
OFFICE OF NAVAJO AND HOPI INDIAN RELOCATION	Trump	9.89	1.54	-8.35
OFFICE OF PERSONNEL MANAGEMENT	Trump	7.26	7.47	0.22
OFFICE OF PERSONNEL MANAGEMENT	Obama	7.05	11.76	4.71
OFFICE OF PERSONNEL MANAGEMENT	Bush	7.97	8.00	0.03
OFFICE OF SCIENCE AND TECHNOLOGY POLICY	Trump	8.15	4.61	-3.54
OFFICE OF SCIENCE AND TECHNOLOGY POLICY	Obama	7.10	5.17	-1.93
OFFICE OF SCIENCE AND TECHNOLOGY POLICY	Bush	14.22	14.65	0.43
OFFICE OF SPECIAL COUNSEL	Trump	12.62	9.50	-3.12
OFFICE OF SPECIAL COUNSEL	Obama	7.28	5.94	-1.35
OFFICE OF SPECIAL COUNSEL	Bush	12.09	8.50	-3.59
OFFICE OF THE U.S. TRADE REPRESENTATIVE	Trump	11.38	14.44	3.07
OFFICE OF THE U.S. TRADE REPRESENTATIVE	Obama	7.89	6.00	-1.89
OFFICE OF THE U.S. TRADE REPRESENTATIVE	Bush	11.62	18.77	7.15
OVERSEAS PRIVATE INVESTMENT CORPORATION	Trump	11.05	15.51	4.46
OVERSEAS PRIVATE INVESTMENT CORPORATION	Obama	10.25	13.26	3.01
OVERSEAS PRIVATE INVESTMENT CORPORATION	Bush	11.87	13.80	1.92
PENSION BENEFIT GUARANTY CORPORATION	Trump	7.88	6.09	-1.80
PENSION BENEFIT GUARANTY CORPORATION	Obama	6.35	7.67	1.32
PENSION BENEFIT GUARANTY CORPORATION	Bush	6.00	6.74	0.75
RAILROAD RETIREMENT BOARD	Trump	7.81	5.61	-2.20
RAILROAD RETIREMENT BOARD	Obama	6.22	6.07	-0.16
RAILROAD RETIREMENT BOARD	Bush	5.12	1.80	-3.32
SECURITIES AND EXCHANGE COMMISSION	Trump	3.80	2.34	-1.47
SECURITIES AND EXCHANGE COMMISSION	Obama	5.56	8.09	2.53
SECURITIES AND EXCHANGE COMMISSION	Bush	6.22	12.88	6.65

Table S.1. Bayesian Adjusted Rates in Agencies *(continued)*

Agency	Pres.	Sep. Rate	Acc. Rate	Net Rate
SELECTIVE SERVICE SYSTEM	Trump	17.91	19.37	1.45
SELECTIVE SERVICE SYSTEM	Obama	12.25	14.11	1.86
SELECTIVE SERVICE SYSTEM	Bush	8.01	4.90	-3.11
SMALL BUSINESS ADMINISTRATION	Trump	9.76	7.96	-1.79
SMALL BUSINESS ADMINISTRATION	Obama	8.55	7.81	-0.75
SMALL BUSINESS ADMINISTRATION	Bush	6.82	1.47	-5.35
SMITHSONIAN INSTITUTION	Trump	8.99	7.71	-1.27
SMITHSONIAN INSTITUTION	Obama	6.81	6.69	-0.13
SMITHSONIAN INSTITUTION	Bush	8.85	8.99	0.14
SOCIAL SECURITY ADMINISTRATION	Trump	6.54	5.71	-0.83
SOCIAL SECURITY ADMINISTRATION	Obama	5.76	5.37	-0.39
SOCIAL SECURITY ADMINISTRATION	Bush	5.39	5.78	0.38
TRADE AND DEVELOPMENT AGENCY	Trump	16.51	18.94	2.42
TRADE AND DEVELOPMENT AGENCY	Obama	10.08	11.60	1.52
TRADE AND DEVELOPMENT AGENCY	Bush	15.10	19.99	4.88
U.S. AGENCY FOR GLOBAL MEDIA	Trump	6.14	3.40	-2.74
U.S. AGENCY FOR GLOBAL MEDIA	Obama	6.54	4.20	-2.34
U.S. AGENCY FOR GLOBAL MEDIA	Bush	5.12	4.16	-0.96
U.S. AGENCY FOR INTERNATIONAL DEVELOPMENT	Trump	6.39	4.76	-1.63
U.S. AGENCY FOR INTERNATIONAL DEVELOPMENT	Obama	8.72	10.84	2.12
U.S. AGENCY FOR INTERNATIONAL DEVELOPMENT	Bush	6.66	4.39	-2.28
U.S. INSTITUTE OF PEACE	Bush	9.04	5.48	-3.56
U.S. INTERNATIONAL TRADE COMMISSION	Trump	10.31	8.63	-1.68
U.S. INTERNATIONAL TRADE COMMISSION	Obama	10.66	9.20	-1.46
U.S. INTERNATIONAL TRADE COMMISSION	Bush	7.00	5.66	-1.34

Table S.2. Bayesian Adjusted Rates in Bureaus of Executive Departments (Trump Administration Only)

Dept.	Bureau	Sep. Rate	Acc. Rate	Net Rate
USDA	OFFICE OF THE SECRETARY OF AGRICULTURE	8.07	8.48	0.41
USDA	AGRICULTURAL MARKETING SERVICE	9.18	6.50	-2.69
USDA	AGRICULTURAL RESEARCH SERVICE	6.90	4.67	-2.23
USDA	RURAL DEVELOPMENT	8.37	5.64	-2.72
USDA	RISK MANAGEMENT AGENCY	7.24	5.36	-1.88
USDA	FOREIGN AGRICULTURAL SERVICE	7.35	6.11	-1.24
USDA	FOREST SERVICE	7.96	5.45	-2.51
USDA	OFFICE OF COMMUNICATIONS	7.89	3.37	-4.52
USDA	OFFICE OF THE GENERAL COUNSEL	9.30	6.88	-2.43
USDA	RURAL UTILITIES SERVICE	5.84	1.96	-3.88
USDA	NATURAL RESOURCES CONSERVATION SERVICE	7.55	7.44	-0.10
USDA	ECONOMIC RESEARCH SERVICE	13.40	9.61	-3.79
USDA	NATIONAL AGRICULTURAL STATISTICS SERVICE	5.78	2.36	-3.42
USDA	NATIONAL INSTITUTE OF FOOD AND AGRICULTURE	12.84	12.69	-0.15
USDA	OFFICE OF THE INSPECTOR GENERAL	8.40	5.85	-2.55
USDA	FOOD AND NUTRITION SERVICE	7.35	3.93	-3.42
USDA	RURAL BUSINESS-COOPERATIVE SERVICE	6.67	2.30	-4.37
USDA	ANIMAL AND PLANT HEALTH INSPECTION SERVICE	7.16	4.91	-2.25
USDA	FOOD SAFETY AND INSPECTION SERVICE	8.16	5.73	-2.42
USDA	OFFICE OF THE CHIEF ECONOMIST	9.19	4.46	-4.72
USDA	OFFICE OF BUDGET AND PROGRAM ANALYSIS	9.21	5.50	-3.71
USDA	OFFICE OF THE CHIEF FINANCIAL OFFICER	7.17	3.09	-4.08
USDA	OFFICE OF ADVOCACY AND OUTREACH	7.28	2.11	-5.17
USDA	CIVIL RIGHTS	7.35	3.33	-4.02
USDA	DEPARTMENTAL ADMINISTRATION	6.41	4.06	-2.35
USDA	FARM SERVICE AGENCY	7.58	5.98	-1.60

Table S.2. Bayesian Adjusted Rates in Bureaus of Executive Departments (Trump Administration Only) *(continued)*

Dept.	Bureau	Sep. Rate	Acc. Rate	Net Rate
USDA	HOMELAND SECURITY STAFF	9.03	9.93	0.89
USDA	OFFICE OF THE CHIEF INFORMATION OFFICER	9.83	7.08	-2.75
USDA	NATIONAL APPEALS DIVISION	9.23	4.93	-4.29
DOC	OFFICE OF THE SECRETARY	11.91	12.81	0.90
DOC	ECONOMIC DEVELOPMENT ADMINISTRATION	7.20	5.75	-1.45
DOC	BUREAU OF ECONOMIC ANALYSIS	6.75	4.62	-2.13
DOC	NATIONAL OCEANIC AND ATMOSPHERIC ADMINISTRATION	6.15	6.30	0.15
DOC	INTERNATIONAL TRADE ADMINISTRATION	7.37	6.27	-1.09
DOC	PATENT AND TRADEMARK OFFICE	3.89	4.82	0.93
DOC	NATIONAL INSTITUTE OF STANDARDS AND TECHNOLOGY	6.09	4.40	-1.69
DOC	MINORITY BUSINESS DEVELOPMENT AGENCY	8.80	4.88	-3.92
DOC	NATIONAL TELECOMMUNICATIONS AND INFORMATION ADMINISTRATION	9.70	7.62	-2.08
DOC	NATIONAL TECHNICAL INFORMATION SERVICE	15.57	4.65	-10.93
DOC	U.S. CENSUS BUREAU	5.49	2.89	-2.60
DOC	OFFICE OF THE INSPECTOR GENERAL	14.86	14.98	0.12
DOC	BUREAU OF INDUSTRY AND SECURITY	6.49	5.03	-1.46
DOD	OFFICE OF THE SECRETARY OF DEFENSE	10.12	8.30	-1.81
DOD	ORGANIZATION OF THE JOINT CHIEFS OF STAFF	9.23	7.53	-1.71
DOD	DEFENSE INFORMATION SYSTEMS AGENCY	9.57	11.39	1.82
DOD	DEFENSE SECURITY COOPERATION AGENCY	13.77	20.51	6.74
DOD	DEFENSE LOGISTICS AGENCY	9.04	7.87	-1.17
DOD	U.S. COURT OF APPEALS FOR THE ARMED FORCES	9.41	6.25	-3.16
DOD	DEFENSE CONTRACT AUDIT AGENCY	8.87	8.70	-0.17
DOD	DEFENSE COUNTERINTELLIGENCE AND SECURITY AGENCY	19.71	18.28	-1.42
DOD	DEFENSE ADVANCED RESEARCH PROJECTS AGENCY	9.71	10.47	0.76
DOD	UNIFORMED SERVICES UNIVERSITY OF THE HEALTH SCIENCES	8.65	7.64	-1.01
DOD	DEPARTMENT OF DEFENSE EDUCATION ACTIVITY	13.21	14.75	1.54

Table S.2. Bayesian Adjusted Rates in Bureaus of Executive Departments (Trump Administration Only) *(continued)*

Dept.	Bureau	Sep. Rate	Acc. Rate	Net Rate
DOD	WASHINGTON HEADQUARTERS SERVICES	9.19	10.26	1.06
DOD	OFFICE OF ECONOMIC ADJUSTMENT	10.79	9.02	-1.77
DOD	DEFENSE LEGAL SERVICES AGENCY	6.10	3.62	-2.48
DOD	OFFICE OF THE INSPECTOR GENERAL	9.58	11.38	1.81
DOD	MISSILE DEFENSE AGENCY	8.50	10.72	2.22
DOD	DEFENSE TECHNOLOGY SECURITY ADMINISTRATION	9.08	8.75	-0.33
DOD	DEFENSE COMMISSARY AGENCY	15.23	4.12	-11.11
DOD	DEFENSE FINANCE AND ACCOUNTING SERVICE	8.34	8.28	-0.06
DOD	DEFENSE HUMAN RESOURCES ACTIVITY	11.00	12.35	1.35
DOD	DEFENSE POW/MIA ACCOUNTING AGENCY	9.73	9.20	-0.53
DOD	DEFENSE HEALTH AGENCY	5.10	6.85	1.74
DOD	DEFENSE THREAT REDUCTION AGENCY	9.44	9.95	0.51
DOD	DEFENSE CONTRACT MANAGEMENT AGENCY	8.45	7.04	-1.40
DOD	PENTAGON FORCE PROTECTION AGENCY	9.07	8.29	-0.78
DOD	DEPARTMENT OF DEFENSE TEST RESOURCE MANAGEMENT CENTER	8.64	4.53	-4.11
DOD	NATIONAL DEFENSE UNIVERSITY	10.90	6.56	-4.34
DOD	DEFENSE MICROELECTRONICS ACTIVITY	10.42	10.44	0.02
DOD	DEFENSE TECHNICAL INFORMATION CENTER	7.78	6.42	-1.36
DOD	DEFENSE MEDIA ACTIVITY	10.59	6.96	-3.64
DOD	DEFENSE ACQUISITION UNIVERSITY	8.05	7.01	-1.05
DOD	NATIONAL RECONNAISSANCE OFFICE	9.18	17.63	8.46
DOD	PARTMENT OF THE AIR FORCE	8.83	8.91	0.08
DOD	PARTMENT OF THE ARMY	9.47	8.26	-1.21
DOD	PARTMENT OF THE NAVY	8.03	9.48	1.46
DOJ	OFFICES, BOARDS AND DIVISIONS	8.35	3.34	-5.02
DOJ	FEDERAL BUREAU OF INVESTIGATION	5.52	4.54	-0.98
DOJ	BUREAU OF PRISONS/FEDERAL PRISON SYSTEM	7.15	5.54	-1.61

Table S.2. Bayesian Adjusted Rates in Bureaus of Executive Departments (Trump Administration Only) *(continued)*

Dept.	Bureau	Sep. Rate	Acc. Rate	Net Rate
DOJ	DRUG ENFORCEMENT ADMINISTRATION	6.36	6.23	-0.12
DOJ	OFFICE OF JUSTICE PROGRAMS	6.49	2.87	-3.62
DOJ	U.S. MARSHALS SERVICE	6.33	6.90	0.57
DOJ	EXECUTIVE OFFICE FOR U.S. ATTORNEYS AND THE OFFICES OF THE U.S. ATTORNEYS	8.06	4.87	-3.18
DOJ	OFFICE OF THE INSPECTOR GENERAL	8.62	6.83	-1.79
DOJ	U.S. TRUSTEE PROGRAM	6.28	2.14	-4.14
DOJ	EXECUTIVE OFFICE FOR IMMIGRATION REVIEW	10.66	14.70	4.04
DOJ	COMMUNITY RELATIONS SERVICE	8.42	5.12	-3.30
DOJ	BUREAU OF ALCOHOL, TOBACCO, FIREARMS, AND EXPLOSIVES	6.22	5.09	-1.13
DOL	OFFICE OF THE SECRETARY OF LABOR	8.11	4.92	-3.19
DOL	OFFICE OF THE ASSISTANT SECRETARY FOR ADMINISTRATION AND MANAGEMENT	9.83	8.34	-1.49
DOL	BUREAU OF INTERNATIONAL LABOR AFFAIRS	8.54	8.72	0.18
DOL	OFFICE OF CONGRESSIONAL AND INTERGOVERNMENTAL AFFAIRS	7.60	4.06	-3.54
DOL	OFFICE OF THE CHIEF FINANCIAL OFFICER	9.13	9.42	0.29
DOL	OFFICE OF DISABILITY EMPLOYMENT POLICY	8.44	6.48	-1.96
DOL	EMPLOYMENT AND TRAINING ADMINISTRATION	6.53	3.08	-3.46
DOL	OFFICE OF THE INSPECTOR GENERAL	7.96	4.30	-3.67
DOL	BUREAU OF LABOR STATISTICS	7.73	5.98	-1.75
DOL	MINE SAFETY AND HEALTH ADMINISTRATION	7.32	2.02	-5.30
DOL	OFFICE OF FEDERAL CONTRACT COMPLIANCE PROGRAMS	7.34	1.82	-5.52
DOL	OFFICE OF LABOR-MANAGEMENT STANDARDS	6.64	4.62	-2.01
DOL	OFFICE OF WORKERS' COMPENSATION PROGRAMS	7.95	4.41	-3.54
DOL	OFFICE OF PUBLIC AFFAIRS	6.67	4.32	-2.36
DOL	OFFICE OF THE ASSISTANT SECRETARY FOR POLICY	7.11	4.80	-2.31
DOL	EMPLOYEE BENEFITS SECURITY ADMINISTRATION	7.17	3.69	-3.48

Table S.2. Bayesian Adjusted Rates in Bureaus of Executive Departments (Trump Administration Only) *(continued)*

Dept.	Bureau	Sep. Rate	Acc. Rate	Net Rate
DOL	OCCUPATIONAL SAFETY AND HEALTH ADMINISTRATION	7.49	5.38	-2.11
DOL	OFFICE OF THE SOLICITOR	7.67	4.99	-2.68
DOL	VETERANS EMPLOYMENT AND TRAINING SERVICES	7.09	4.91	-2.19
DOL	WOMEN'S BUREAU	8.42	4.50	-3.91
DOL	WAGE AND HOUR DIVISION	6.82	3.23	-3.60
ED	IMMEDIATE OFFICE OF THE SECRETARY OF EDUCATION	8.30	6.06	-2.24
ED	OFFICE FOR CIVIL RIGHTS	7.38	6.55	-0.83
ED	OFFICE OF PLANNING, EVALUATION AND POLICY DEVELOPMENT	7.66	4.58	-3.07
ED	OFFICE OF THE UNDER SECRETARY	7.63	3.04	-4.59
ED	OFFICE OF INSPECTOR GENERAL	7.86	5.23	-2.63
ED	OFFICE OF THE GENERAL COUNSEL	8.24	5.64	-2.60
ED	OFFICE OF SPECIAL EDUCATION AND REHABILITATIVE SERVICES	7.39	4.10	-3.30
ED	OFFICE OF THE CHIEF INFORMATION OFFICER	8.80	8.60	-0.20
ED	OFFICE OF LEGISLATION AND CONGRESSIONAL AFFAIRS	8.19	4.92	-3.27
ED	FEDERAL STUDENT AID	7.32	6.90	-0.42
ED	OFFICE OF COMMUNICATIONS AND OUTREACH	7.28	2.67	-4.61
ED	OFFICE OF POSTSECONDARY EDUCATION	7.31	3.06	-4.24
ED	INSTITUTE OF EDUCATION SCIENCES	6.49	1.75	-4.74
ED	OFFICE OF ELEMENTARY AND SECONDARY EDUCATION	9.19	6.94	-2.25
ED	OFFICE OF ENGLISH LANGUAGE ACQUISITION	8.05	7.16	-0.89
ED	OFFICE OF CAREER, TECHNICAL, AND ADULT EDUCATION	7.69	3.60	-4.09
ED	NATIONAL ASSESSMENT GOVERNING BOARD	7.78	4.69	-3.09
HHS	OFFICE OF THE SECRETARY OF HEALTH AND HUMAN SERVICES	7.25	8.39	1.13
HHS	PROGRAM SUPPORT CENTER	10.60	6.92	-3.67
HHS	ADMINISTRATION FOR COMMUNITY LIVING	9.18	6.20	-2.98
HHS	OFFICE OF INSPECTOR GENERAL	6.22	5.94	-0.28

Table S.2. Bayesian Adjusted Rates in Bureaus of Executive Departments (Trump Administration Only) *(continued)*

Dept.	Bureau	Sep. Rate	Acc. Rate	Net Rate
HHS	SUBSTANCE ABUSE AND MENTAL HEALTH SERVICES ADMINISTRATION	8.83	7.69	-1.14
HHS	AGENCY FOR HEALTHCARE RESEARCH AND QUALITY	5.27	2.13	-3.14
HHS	HEALTH RESOURCES AND SERVICES ADMINISTRATION	5.97	5.75	-0.22
HHS	AGENCY FOR TOXIC SUBSTANCES AND DISEASE REGISTRY	6.41	1.77	-4.64
HHS	FOOD AND DRUG ADMINISTRATION	5.05	5.68	0.64
HHS	INDIAN HEALTH SERVICE	12.07	12.28	0.21
HHS	NATIONAL INSTITUTES OF HEALTH	5.62	6.21	0.59
HHS	CENTERS FOR DISEASE CONTROL AND PREVENTION	5.71	5.40	-0.31
HHS	CENTERS FOR MEDICARE & MEDICAID SERVICES	5.28	4.32	-0.96
HHS	ADMINISTRATION FOR CHILDREN AND FAMILIES	6.77	7.31	0.54
DHS	DHS HEADQUARTERS	11.94	11.94	0.00
DHS	CITIZENSHIP AND IMMIGRATION SERVICES	6.55	10.27	3.72
DHS	U.S. COAST GUARD	9.06	10.20	1.14
DHS	U.S. SECRET SERVICE	6.52	2.85	-3.67
DHS	OFFICE OF THE INSPECTOR GENERAL	9.41	8.01	-1.40
DHS	IMMIGRATION AND CUSTOMS ENFORCEMENT	5.80	5.62	-0.18
DHS	TRANSPORTATION SECURITY ADMINISTRATION	10.48	7.11	-3.37
DHS	CUSTOMS AND BORDER PROTECTION	4.29	5.94	1.66
DHS	FEDERAL LAW ENFORCEMENT TRAINING CENTERS	7.78	5.53	-2.25
DHS	CYBERSECURITY AND INFRASTRUCTURE SECURITY AGENCY	7.10	8.65	1.55
DHS	FEDERAL EMERGENCY MANAGEMENT AGENCY	8.20	7.12	-1.08
DHS	DOMESTIC NUCLEAR DETECTION OFFICE	11.52	16.19	4.67
DHS	SCIENCE AND TECHNOLOGY DIRECTORATE	7.71	5.95	-1.76
HUD	OFFICE OF THE SENIOR COORDINATOR FOR NEW ENGLAND	7.32	7.34	0.02
HUD	OFFICE OF THE SENIOR COORDINATOR FOR NEW YORK/NEW JERSEY	7.32	7.13	-0.19
HUD	OFFICE OF THE SENIOR COORDINATOR FOR MID-ATLANTIC	7.07	5.43	-1.64
HUD	OFFICE OF THE SENIOR COORDINATOR FOR SOUTHEAST/CARIBBEAN	7.35	5.56	-1.79

Table S.2. Bayesian Adjusted Rates in Bureaus of Executive Departments (Trump Administration Only) *(continued)*

Dept.	Bureau	Sep. Rate	Acc. Rate	Net Rate
HUD	OFFICE OF THE SENIOR COORDINATOR FOR MIDWEST	6.92	6.49	-0.43
HUD	OFFICE OF THE SENIOR COORDINATOR FOR SOUTHWEST	7.97	5.77	-2.20
HUD	OFFICE OF THE SENIOR COORDINATOR FOR GREAT PLAINS	7.54	7.78	0.24
HUD	OFFICE OF THE SENIOR COORDINATOR FOR ROCKY MOUNTAINS	7.41	6.66	-0.74
HUD	OFFICE OF THE SENIOR COORDINATOR FOR PACIFIC/HAWAII	6.85	4.82	-2.03
HUD	OFFICE OF THE SENIOR COORDINATOR FOR NORTHWEST/ALASKA	7.49	7.30	-0.19
HUD	OFFICE OF THE SECRETARY OF HOUSING AND URBAN DEVELOPMENT	7.23	4.45	-2.78
HUD	OFFICE OF THE CHIEF HUMAN CAPITAL OFFICER	8.45	7.71	-0.74
HUD	OFFICE OF GENERAL COUNSEL	7.21	7.30	0.09
HUD	ASSISTANT SECRETARY FOR COMMUNITY PLANNING AND DEVELOPMENT	7.67	7.44	-0.23
HUD	ASSISTANT SECRETARY FOR FAIR HOUSING AND EQUAL OPPORTUNITY	7.34	4.02	-3.32
HUD	OFFICE OF THE CHIEF FINANCIAL OFFICER	8.10	8.89	0.79
HUD	OFFICE OF INSPECTOR GENERAL	7.01	3.01	-4.00
HUD	ASSISTANT SECRETARY FOR CONGRESSIONAL AND INTERGOVERNMENTAL RELATIONS	7.48	5.38	-2.10
HUD	CENTER FOR FAITH-BASED AND COMMUNITY INITIATIVES	7.37	5.40	-1.97
HUD	OFFICE OF FIELD POLICY AND MANAGEMENT	7.45	8.18	0.73
HUD	OFFICE OF HEALTHY HOMES AND LEAD HAZARD CONTROL	6.94	5.23	-1.71
HUD	ASSISTANT SECRETARY FOR HOUSING–FEDERAL HOUSING COMMISSIONER	6.75	5.10	-1.65
HUD	OFFICE OF THE CHIEF PROCUREMENT OFFICER	8.44	9.55	1.11
HUD	ASSISTANT SECRETARY FOR PUBLIC AND INDIAN HOUSING	7.30	5.81	-1.50
HUD	OFFICE OF THE CHIEF INFORMATION OFFICER	8.15	5.96	-2.19
HUD	ASSISTANT SECRETARY FOR POLICY DEVELOPMENT AND RESEARCH	7.04	6.67	-0.37
HUD	GOVERNMENT NATIONAL MORTGAGE ASSOCIATION (GINNIE MAE)	7.21	6.23	-0.98
HUD	OFFICE OF DEPARTMENTAL EQUAL EMPLOYMENT OPPORTUNITY	8.15	8.56	0.40
HUD	ASSISTANT SECRETARY FOR PUBLIC AFFAIRS	7.56	4.62	-2.94
HUD	OFFICE OF STRATEGIC PLANNING AND MANAGEMENT	7.33	4.91	-2.42

Table S.2. Bayesian Adjusted Rates in Bureaus of Executive Departments (Trump Administration Only) *(continued)*

Dept.	Bureau	Sep. Rate	Acc. Rate	Net Rate
DOI	OFFICE OF THE SECRETARY OF THE INTERIOR	8.31	5.22	-3.09
DOI	BUREAU OF LAND MANAGEMENT	8.86	6.98	-1.89
DOI	INDIAN AFFAIRS	8.62	6.32	-2.31
DOI	BUREAU OF RECLAMATION	9.75	8.87	-0.88
DOI	GEOLOGICAL SURVEY	7.05	4.51	-2.53
DOI	NATIONAL PARK SERVICE	7.90	5.07	-2.83
DOI	U.S. FISH AND WILDLIFE SERVICE	7.03	4.63	-2.40
DOI	OFFICE OF THE SOLICITOR	8.84	7.49	-1.35
DOI	OFFICE OF SURFACE MINING, RECLAMATION AND ENFORCEMENT	8.78	4.19	-4.59
DOI	OFFICE OF THE INSPECTOR GENERAL	7.53	6.97	-0.56
DOI	BUREAU OF SAFETY AND ENVIRONMENTAL ENFORCEMENT	6.92	4.20	-2.72
DOI	BUREAU OF OCEAN ENERGY MANAGEMENT	6.44	5.43	-1.01
DOT	OFFICE OF THE SECRETARY OF TRANSPORTATION	8.69	7.79	-0.89
DOT	FEDERAL AVIATION ADMINISTRATION	5.55	3.43	-2.12
DOT	FEDERAL HIGHWAY ADMINISTRATION	7.27	7.31	0.04
DOT	FEDERAL RAILROAD ADMINISTRATION	6.64	5.33	-1.30
DOT	SAINT LAWRENCE SEAWAY DEVELOPMENT CORPORATION	9.66	8.14	-1.52
DOT	FEDERAL TRANSIT ADMINISTRATION	8.71	9.57	0.86
DOT	NATIONAL HIGHWAY TRAFFIC SAFETY ADMINISTRATION	8.00	10.92	2.92
DOT	OFFICE OF INSPECTOR GENERAL	7.57	4.22	-3.35
DOT	MARITIME ADMINISTRATION	7.76	8.10	0.33
DOT	PIPELINE AND HAZARDOUS MATERIALS SAFETY ADMINISTRATION	7.10	7.69	0.60
DOT	FEDERAL MOTOR CARRIER SAFETY ADMINISTRATION	6.89	6.15	-0.74
TREAS	SPECIAL INSPECTOR GENERAL FOR THE TROUBLED ASSETS RELIEF PROGRAM (TARP)	21.45	6.41	-15.04
TREAS	ALCOHOL AND TOBACCO TAX AND TRADE BUREAU	4.78	3.80	-0.98
TREAS	DEPARTMENTAL OFFICES	11.40	10.05	-1.34

Table S.2. Bayesian Adjusted Rates in Bureaus of Executive Departments (Trump Administration Only) *(continued)*

Dept.	Bureau	Sep. Rate	Acc. Rate	Net Rate
TREAS	INTERNAL REVENUE SERVICE	7.04	2.91	-4.13
TREAS	OFFICE OF INSPECTOR GENERAL	8.48	11.50	3.02
TREAS	U.S. MINT	8.23	6.72	-1.51
TREAS	FINANCIAL CRIMES ENFORCEMENT NETWORK	10.71	8.72	-1.99
TREAS	BUREAU OF ENGRAVING AND PRINTING	6.76	6.14	-0.62
TREAS	OFFICE OF THE COMPTROLLER OF THE CURRENCY	7.16	4.25	-2.92
TREAS	BUREAU OF THE FISCAL SERVICE	5.55	5.92	0.38
TREAS	OFFICE OF THE INSPECTOR GENERAL FOR TAX ADMINISTRATION	7.67	4.65	-3.02
VA	OFFICE OF THE SECRETARY	7.91	11.10	3.19
VA	BOARD OF VETERANS APPEALS	11.85	22.67	10.81
VA	GENERAL COUNSEL	6.97	9.69	2.72
VA	INSPECTOR GENERAL	9.52	13.98	4.47
VA	IMMEDIATE OFFICE OF THE ASSISTANT SECRETARY FOR HUMAN RESOURCES AND ADMINISTRATION	9.91	18.82	8.92
VA	CHIEF HUMAN CAPITAL OFFICER	9.60	11.65	2.04
VA	ASSISTANT SECRETARY FOR DIVERSITY & INCLUSION	7.16	4.12	-3.04
VA	DEPUTY ASSISTANT SECRETARY FOR ADMINISTRATION	6.43	7.76	1.32
VA	DEPUTY ASSISTANT SECRETARY FOR OFFICE OF RESOLUTION MANAGEMENT	9.66	7.91	-1.75
VA	DEPUTY ASSISTANT SECRETARY FOR CORPORATE SENIOR EXECUTIVE MANAGEMENT	10.52	13.07	2.55
VA	DEPUTY ASSISTANT SECRETARY FOR LABOR MANAGEMENT RELATIONS	8.47	8.91	0.44
VA	OFFICE OF ENTERPRISE OPERATIONS	7.92	9.49	1.57
VA	IMMEDIATE OFFICE OF THE ASSISTANT SECRETARY FOR MANAGEMENT	9.08	9.73	0.65
VA	DEPUTY ASSISTANT SECRETARY FOR BUDGET	11.55	14.91	3.36
VA	DEPUTY ASSISTANT SECRETARY FOR FINANCE	7.22	14.40	7.18
VA	EXECUTIVE DIRECTOR, OFFICE OF ACQUISITION AND LOGISTICS	7.61	10.89	3.28

Table S.2. Bayesian Adjusted Rates in Bureaus of Executive Departments (Trump Administration Only) *(continued)*

Dept.	Bureau	Sep. Rate	Acc. Rate	Net Rate
VA	IMMEDIATE OFFICE OF THE ASSISTANT SECRETARY FOR INFORMATION AND TECHNOLOGY	8.93	13.06	4.13
VA	DEPUTY ASSISTANT SECRETARY FOR INFORMATION AND TECHNOLOGY	5.45	5.48	0.03
VA	IMMEDIATE OFFICE OF THE ASSISTANT SECRETARY FOR OPERATIONS, SECURITY, AND PREPAREDNESS	7.83	13.28	5.45
VA	EXECUTIVE DIRECTOR FOR SECURITY AND LAW ENFORCEMENT	8.13	5.12	-3.01
VA	DEP ASST SEC FOR EMERGENCY MGT & RESILIENCE	6.42	3.46	-2.96
VA	OFFICE OF ENTERPRISE INTEGRATION	6.53	4.01	-2.53
VA	DEPUTY ASSISTANT SECRETARY FOR PLANNING AND EVALUATION	8.27	7.18	-1.09
VA	VET CUSTOMER EXPER (VCE)	9.07	7.02	-2.05
VA	OFFICE OF THE ASSISTANT SECRETARY FOR PUBLIC AND INTERGOVERNMENTAL AFFAIRS	6.49	3.78	-2.71
VA	DEPUTY ASSISTANCE SECRETARY FOR INTERGOVERNMENTAL AFFAIRS	7.60	5.23	-2.36
VA	DEPUTY ASSISTANT SECRETARY FOR PUBLIC AFFAIRS	8.55	11.17	2.62
VA	IMMEDIATE OFFICE OF THE ASSISTANT SECRETARY FOR CONGRESSIONAL AND LEGISLATIVE AFFAIRS	8.69	8.29	-0.41
VA	DEPUTY ASSISTANT SECRETARY FOR CONGRESSIONAL AFFAIRS	10.19	12.86	2.67
VA	VETERANS BENEFITS ADMINISTRATION	7.91	9.40	1.49
VA	NATIONAL CEMETERY ADMINISTRATION	11.20	11.16	-0.04
VA	VETERANS HEALTH ADMINISTRATION	8.71	11.62	2.91

S3 List of Agencies Excluded from the Analysis

Table S3 contains agencies that were excluded from analysis because we do not believe that their separation and accession rates share a common distribution with other federal agencies. The Peace Corps was excluded because only 1 of its employees is classified as a permanent career civil servant, which is our population of interest.

Table S.3. Agencies Exlcuded from Analysis (Trump Administration)

Agency
AB-AMERICAN BATTLE MONUMENTS COMMISSION
AB-AMERICAN BATTLE MONUMENTS COMMISSION
AP-APPALACHIAN REGIONAL COMMISSION
AW-ARCTIC RESEARCH COMMISSION
BH-COMMISSION FOR THE PRESERVATION OF AMERICA'S HERITAGE ABROAD
BK-JAMES MADISON MEMORIAL FELLOWSHIP FOUNDATION
BT-ARCHITECTURAL AND TRANSPORTATION BARRIERS COMPLIANCE BOARD
BW-NUCLEAR WASTE TECHNICAL REVIEW BOARD
BZ-CHRISTOPHER COLUMBUS FELLOWSHIP FOUNDATION
CF-COMMISSION OF FINE ARTS
CX-NATIONAL COMMISSION ON LIBRARIES AND INFORMATION SCIENCE
DB-PUBLIC INTEREST DECLASSIFICATION BOARD
DG-NORTHERN BORDER REGIONAL COMMISSION
DQ-DENALI COMMISSION
EO-MORRIS K. UDALL AND STEWART L. UDALL FOUNDATION
FQ-COURT SERVICES AND OFFENDER SUPERVISION AGENCY FOR THE DISTRICT OF COLUMBIA
FQ-COURT SERVICES AND OFFENDER SUPERVISION AGENCY FOR THE DISTRICT OF COLUMBIA
GE-BARRY GOLDWATER SCHOLARSHIP AND EXCELLENCE IN EDUCATION FOUNDATION
GJ-PRESIDIO TRUST
GM-VALLES CALDERA TRUST
GO-VIETNAM EDUCATION FOUNDATION
GW-INTERNATIONAL BOUNDARY AND WATER COMMISSION: UNITED STATES AND MEXICO
GX-INTERNATIONAL BOUNDARY COMMISSION: UNITED STATES AND CANADA
GY-INTERNATIONAL JOINT COMMISSION: UNITED STATES AND CANADA
HB-COMMITTEE FOR PURCHASE FROM PEOPLE WHO ARE BLIND OR SE VERELY DISABLED
HD-U.S. HOLOCAUST MEMORIAL MUSEUM
HP-ADVISORY COUNCIL ON HISTORIC PRESERVATION
HT-HARRY S. TRUMAN SCHOLARSHIP FOUNDATION
HW-U.S. INTERAGENCY COUNCIL ON HOMELESSNESS
IG-COUNCIL OF THE INSPECTORS GENERAL ON INTEGRITY AND EFFICIENCY

Table S.3. Agencies Exlcuded from Analysis (Trump Administration) *(continued)*

Agency
JL-JUDICIAL BRANCH
MA-MARINE MAMMAL COMMISSION
NK-NATIONAL COUNCIL ON DISABILITY
NM-NATIONAL MEDIATION BOARD
NP-NATIONAL CAPITAL PLANNING COMMISSION
PU-PEACE CORPS
RH-ARMED FORCES RETIREMENT HOME
RO-MEDICAID AND CHIP PAYMENT AND ACCESS COMMISSION
UJ-JAPAN-UNITED STATES FRIENDSHIP COMMISSION
UT-UTAH RECLAMATION MITIGATION AND CONSERVATION COMMISSION
VD-PRIVACY AND CIVIL LIBERTIES OVERSIGHT BOARD
WX-WORLD WAR 1 CENTENNIAL COMMISSION
ZP-U.S. COMMISSION ON INTERNATIONAL RELIGIOUS FREEDOM
ZS-UNITED STATES-CHINA ECONOMIC AND SECURITY REVIEW COMMISSION
ZU-DWIGHT D. EISENHOWER MEMORIAL COMMISSION

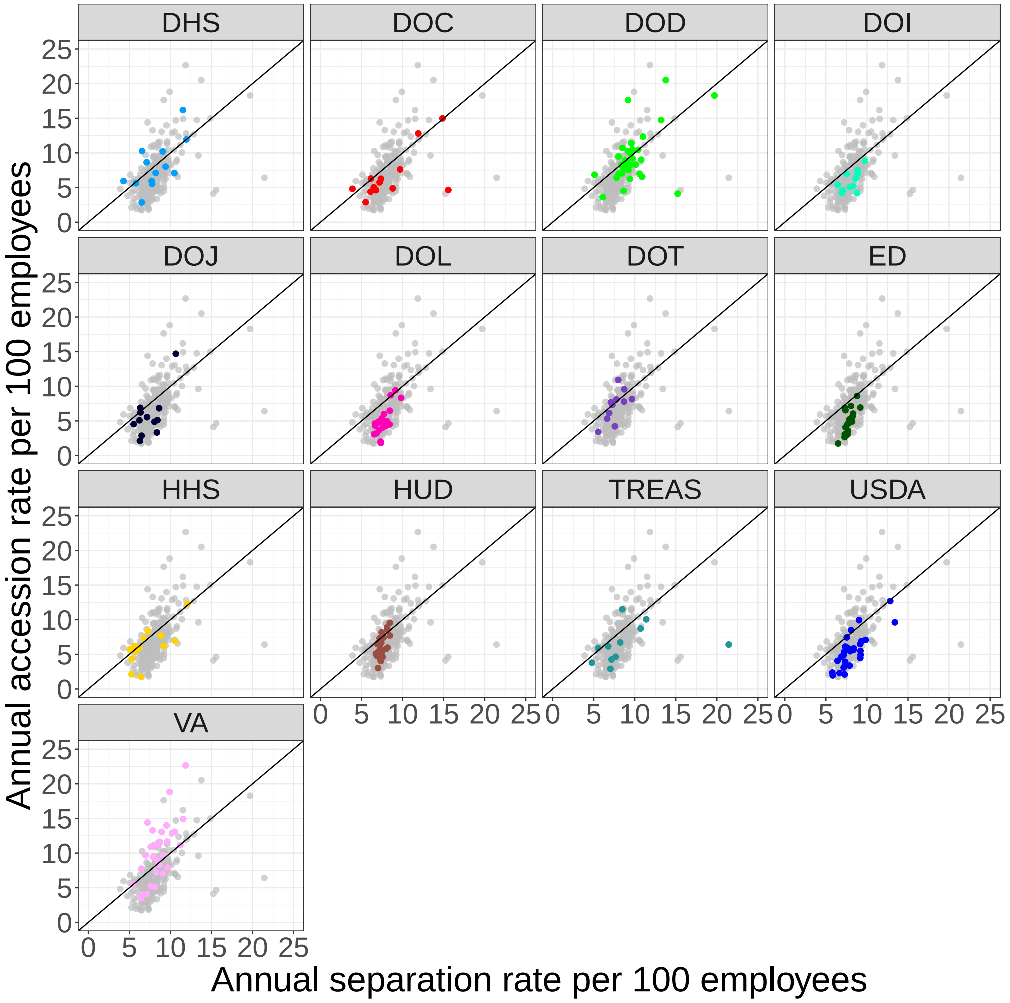

Fig S.1. Separations and Accessions by Department

S4 Small Multiples Plot including Department of Transportation

S5 Ridgeline Plots of Accessions and Separations for Each Executive Department during the Trump Administratoin

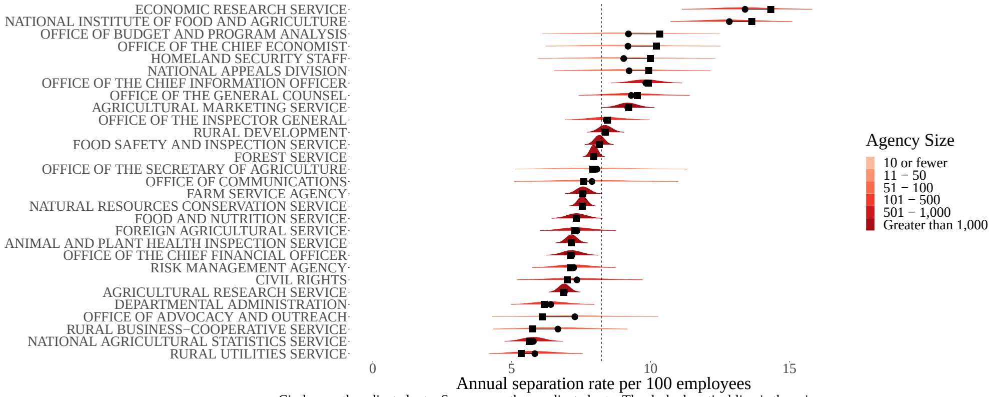

Circles are the adjusted rate. Squares are the unadjusted rate. The dashed vertical line is the prior mean.

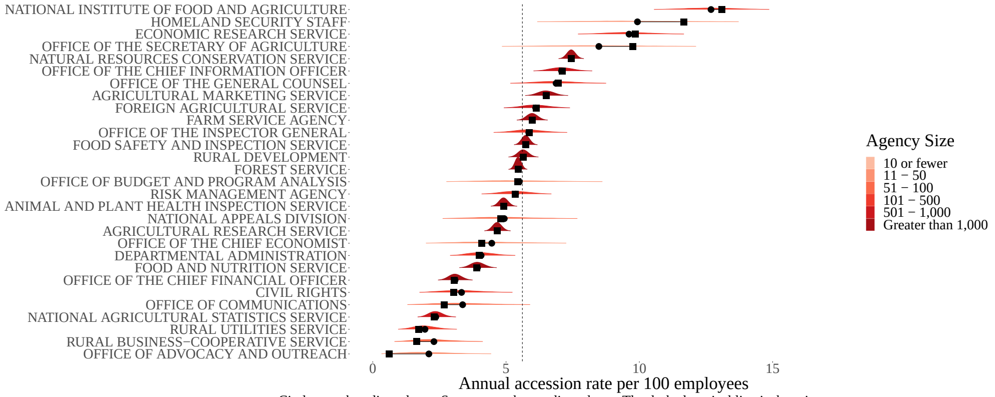

Circles are the adjusted rate. Squares are the unadjusted rate. The dashed vertical line is the prior mean.

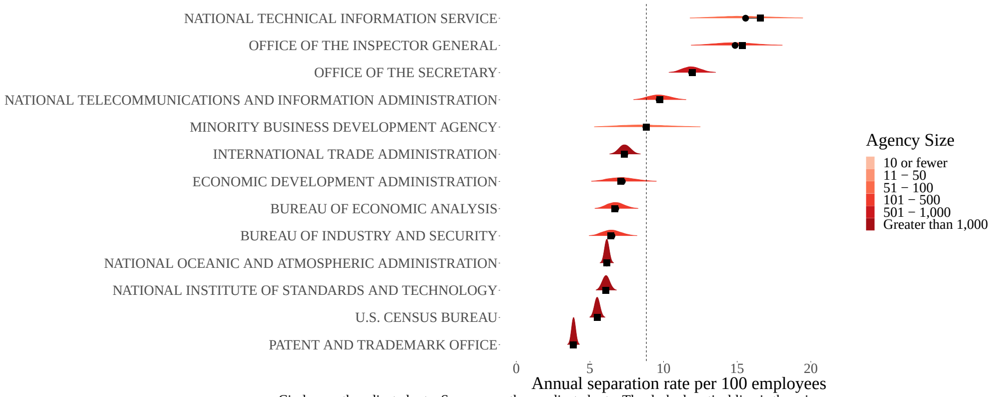

Circles are the adjusted rate. Squares are the unadjusted rate. The dashed vertical line is the prior mean.

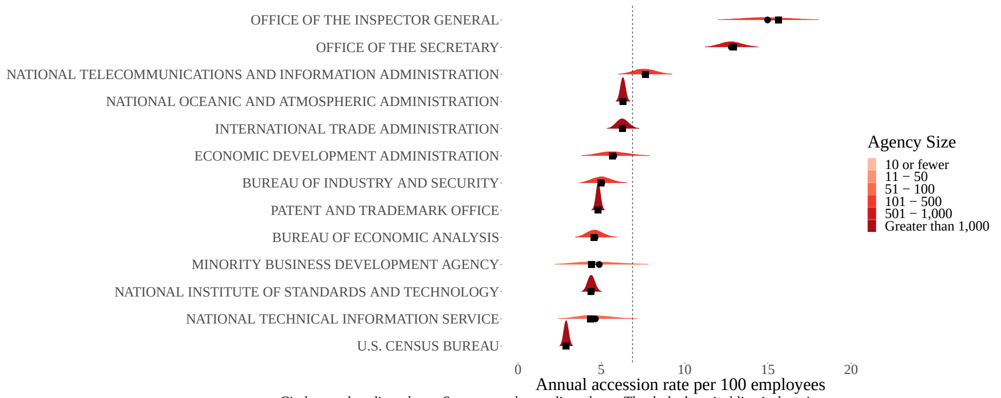

Circles are the adjusted rate. Squares are the unadjusted rate. The dashed vertical line is the prior mean.

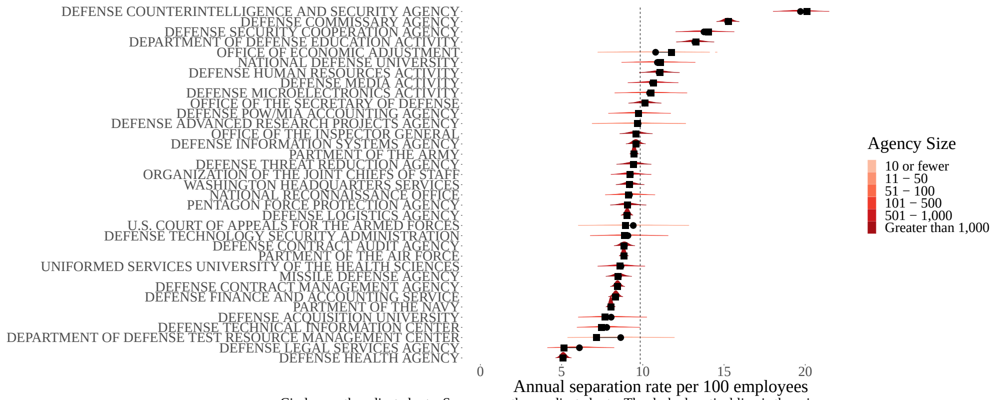

Circles are the adjusted rate. Squares are the unadjusted rate. The dashed vertical line is the prior mean.

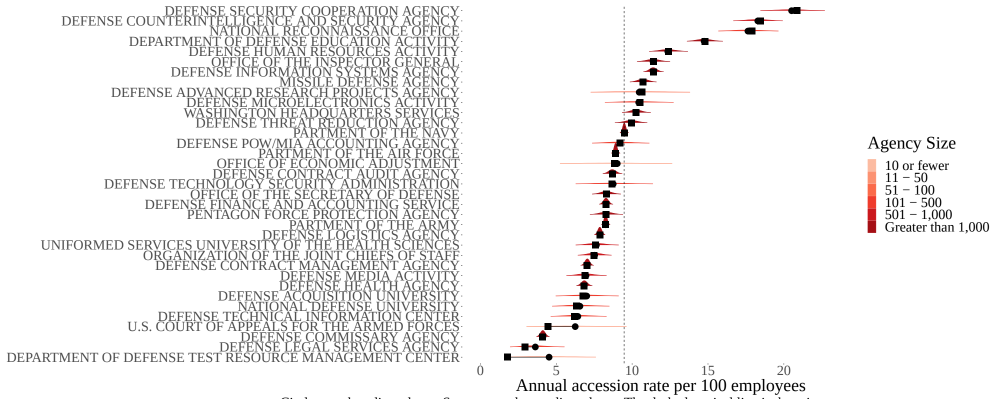

Circles are the adjusted rate. Squares are the unadjusted rate. The dashed vertical line is the prior mean.

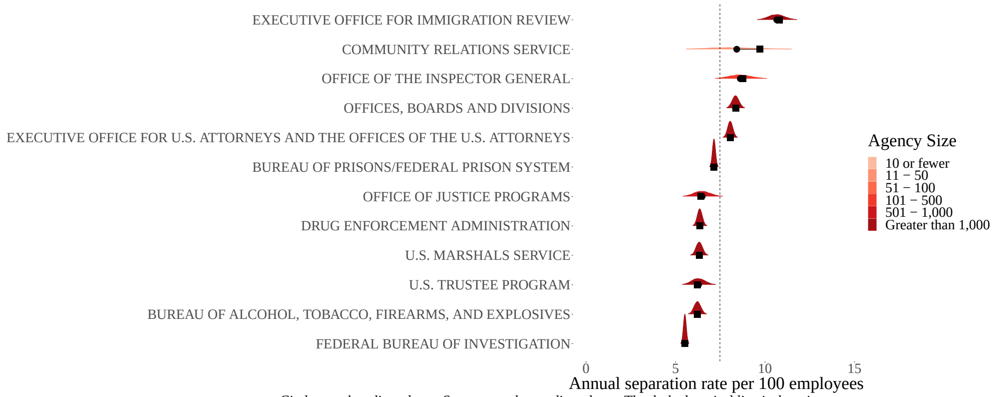

Circles are the adjusted rate. Squares are the unadjusted rate. The dashed vertical line is the prior mean.

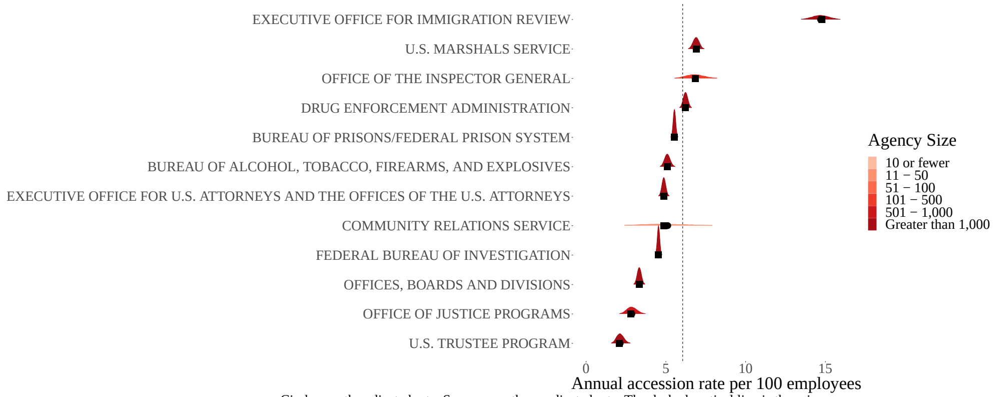

Circles are the adjusted rate. Squares are the unadjusted rate. The dashed vertical line is the prior mean.

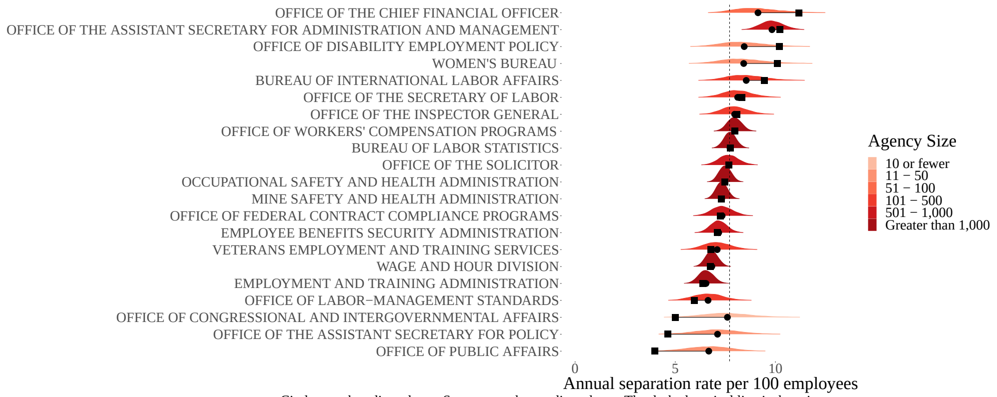

Circles are the adjusted rate. Squares are the unadjusted rate. The dashed vertical line is the prior mean.

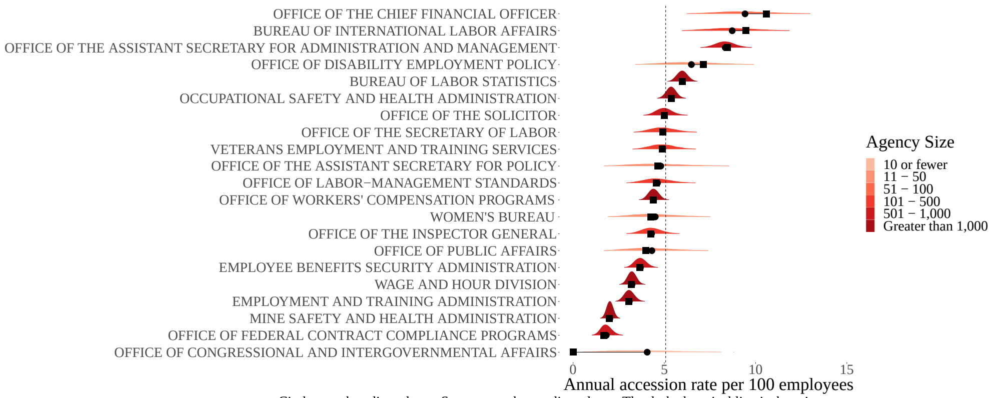

Circles are the adjusted rate. Squares are the unadjusted rate. The dashed vertical line is the prior mean.

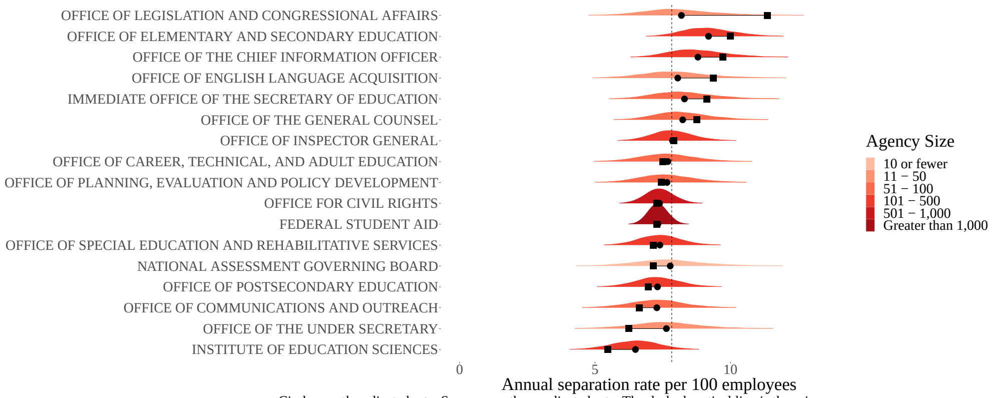

Circles are the adjusted rate. Squares are the unadjusted rate. The dashed vertical line is the prior mean.

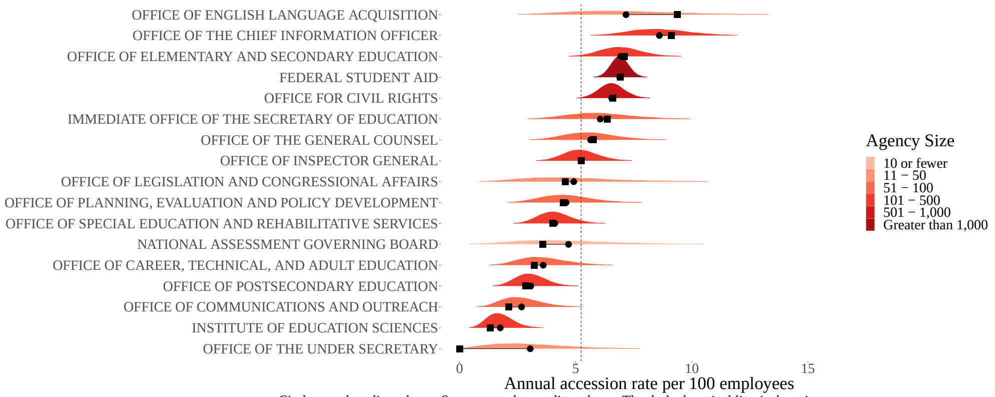

Circles are the adjusted rate. Squares are the unadjusted rate. The dashed vertical line is the prior mean.

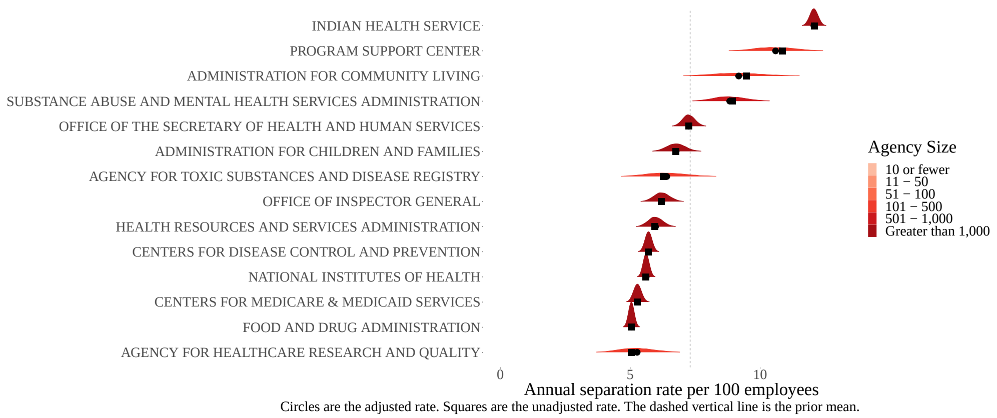

Circles are the adjusted rate. Squares are the unadjusted rate. The dashed vertical line is the prior mean.

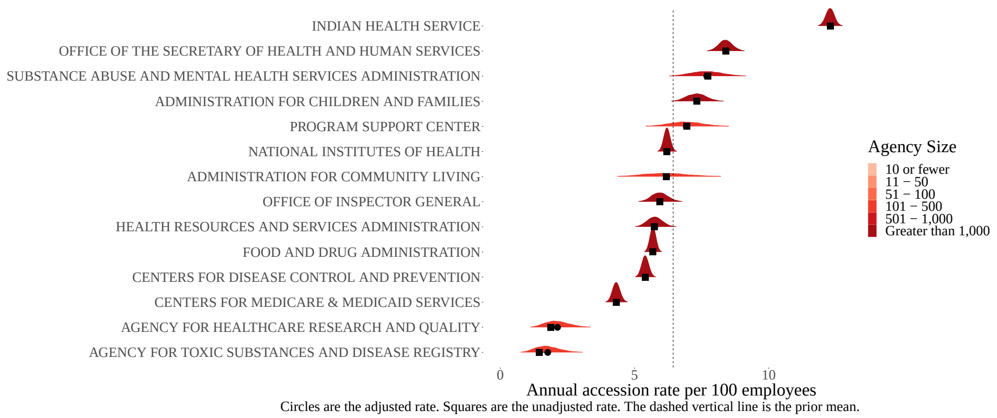

Circles are the adjusted rate. Squares are the unadjusted rate. The dashed vertical line is the prior mean.

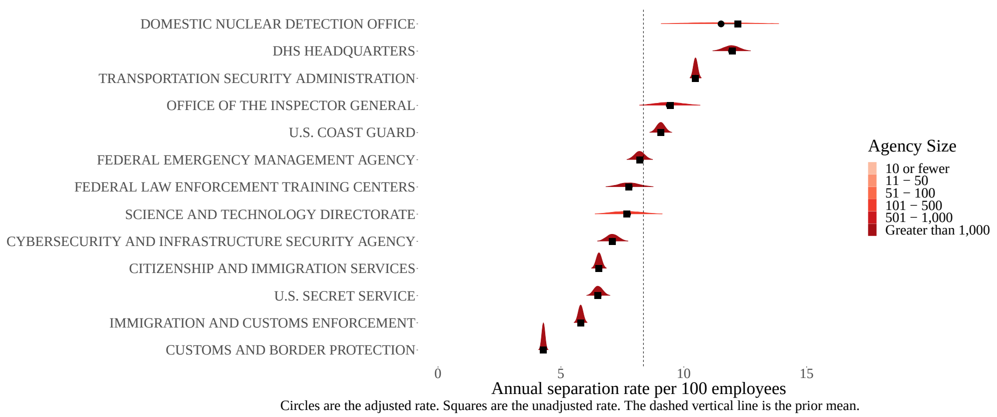

Circles are the adjusted rate. Squares are the unadjusted rate. The dashed vertical line is the prior mean.

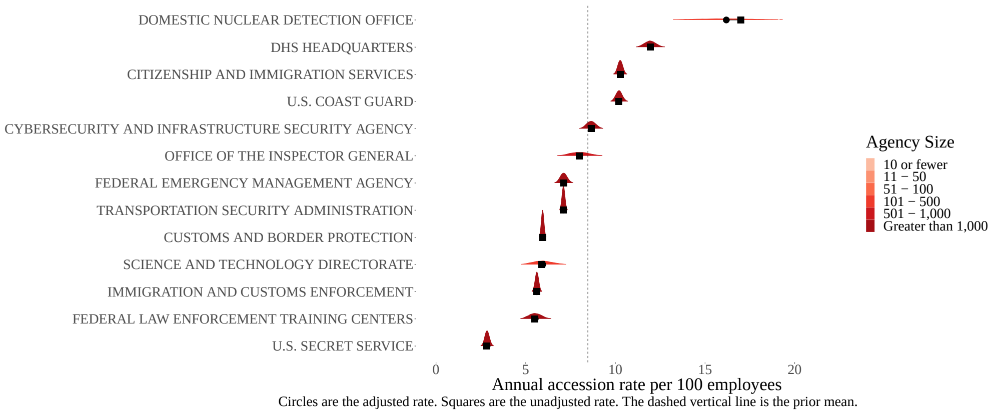

Circles are the adjusted rate. Squares are the unadjusted rate. The dashed vertical line is the prior mean.

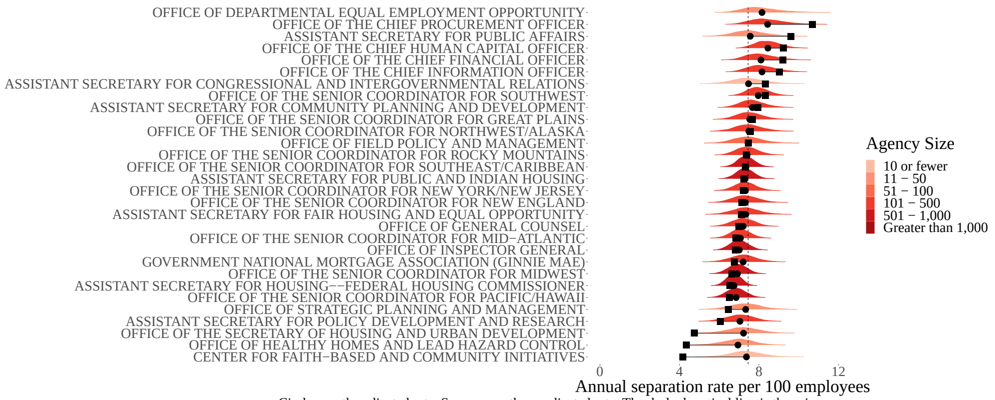

Circles are the adjusted rate. Squares are the unadjusted rate. The dashed vertical line is the prior mean.

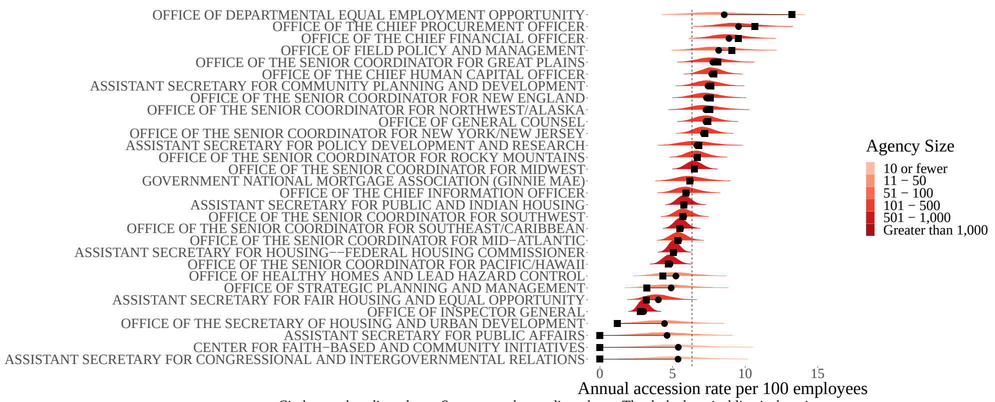

Circles are the adjusted rate. Squares are the unadjusted rate. The dashed vertical line is the prior mean.

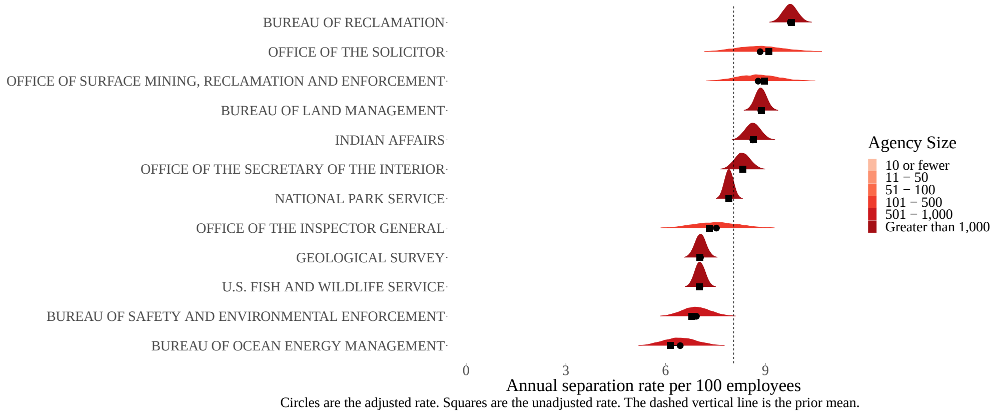
BUREAU OF RECLAMATION
OFFICE OF THE SOLICITOR
OFFICE OF SURFACE MINING, RECLAMATION AND ENFORCEMENT
BUREAU OF LAND MANAGEMENT
INDIAN AFFAIRS
OFFICE OF THE SECRETARY OF THE INTERIOR
NATIONAL PARK SERVICE
OFFICE OF THE INSPECTOR GENERAL
GEOLOGICAL SURVEY
U.S. FISH AND WILDLIFE SERVICE
BUREAU OF SAFETY AND ENVIRONMENTAL ENFORCEMENT
BUREAU OF OCEAN ENERGY MANAGEMENT
0
3
6
9
Annual separation rate per 100 employees
Circles are the adjusted rate. Squares are the unadjusted rate. The dashed vertical line is the prior mean.
Agency Size
10 or fewer
11 − 50
51 − 100
101 − 500
501 − 1,000
Greater than 1,000

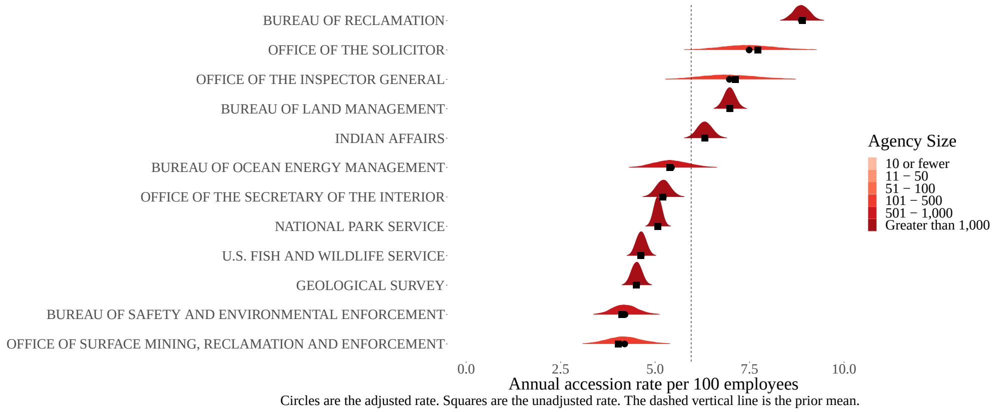
BUREAU OF RECLAMATION
OFFICE OF THE SOLICITOR
OFFICE OF THE INSPECTOR GENERAL
BUREAU OF LAND MANAGEMENT
INDIAN AFFAIRS
BUREAU OF OCEAN ENERGY MANAGEMENT
OFFICE OF THE SECRETARY OF THE INTERIOR
NATIONAL PARK SERVICE
U.S. FISH AND WILDLIFE SERVICE
GEOLOGICAL SURVEY
BUREAU OF SAFETY AND ENVIRONMENTAL ENFORCEMENT
OFFICE OF SURFACE MINING, RECLAMATION AND ENFORCEMENT
0.0
2.5
5.0
7.5
10.0
Annual accession rate per 100 employees
Circles are the adjusted rate. Squares are the unadjusted rate. The dashed vertical line is the prior mean.
Agency Size
10 or fewer
11 − 50
51 − 100
101 − 500
501 − 1,000
Greater than 1,000

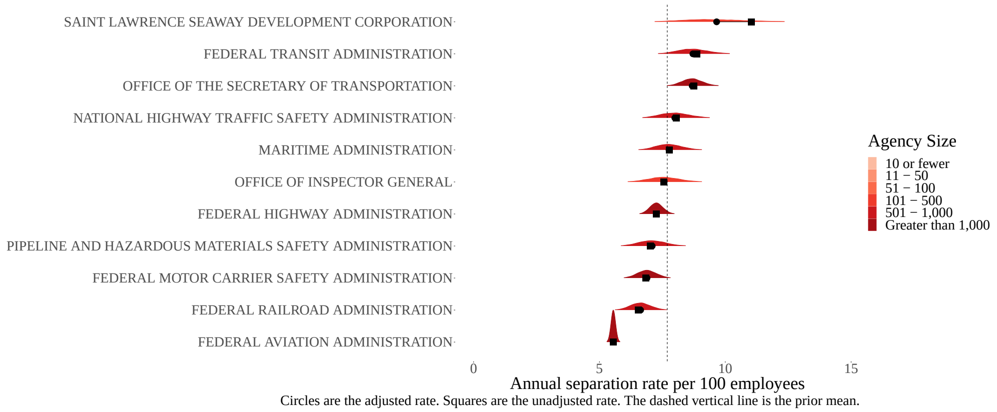
SAINT LAWRENCE SEAWAY DEVELOPMENT CORPORATION
FEDERAL TRANSIT ADMINISTRATION
OFFICE OF THE SECRETARY OF TRANSPORTATION
NATIONAL HIGHWAY TRAFFIC SAFETY ADMINISTRATION
MARITIME ADMINISTRATION
OFFICE OF INSPECTOR GENERAL
FEDERAL HIGHWAY ADMINISTRATION
PIPELINE AND HAZARDOUS MATERIALS SAFETY ADMINISTRATION
FEDERAL MOTOR CARRIER SAFETY ADMINISTRATION
FEDERAL RAILROAD ADMINISTRATION
FEDERAL AVIATION ADMINISTRATION
0
5
10
15
Annual separation rate per 100 employees
Circles are the adjusted rate. Squares are the unadjusted rate. The dashed vertical line is the prior mean.
Agency Size
10 or fewer
11 − 50
51 − 100
101 − 500
501 − 1,000
Greater than 1,000

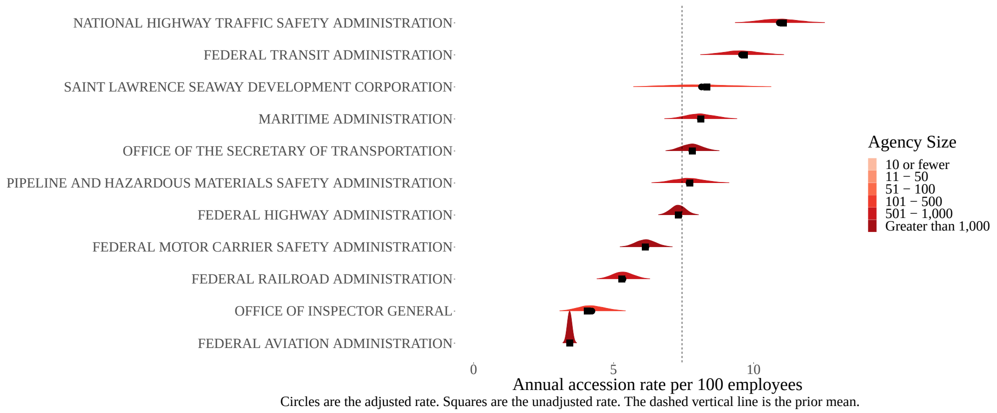

Circles are the adjusted rate. Squares are the unadjusted rate. The dashed vertical line is the prior mean.

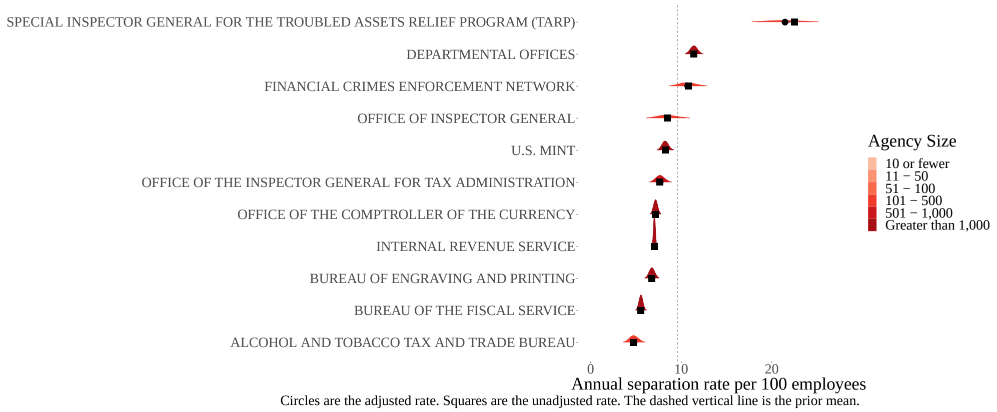

Circles are the adjusted rate. Squares are the unadjusted rate. The dashed vertical line is the prior mean.

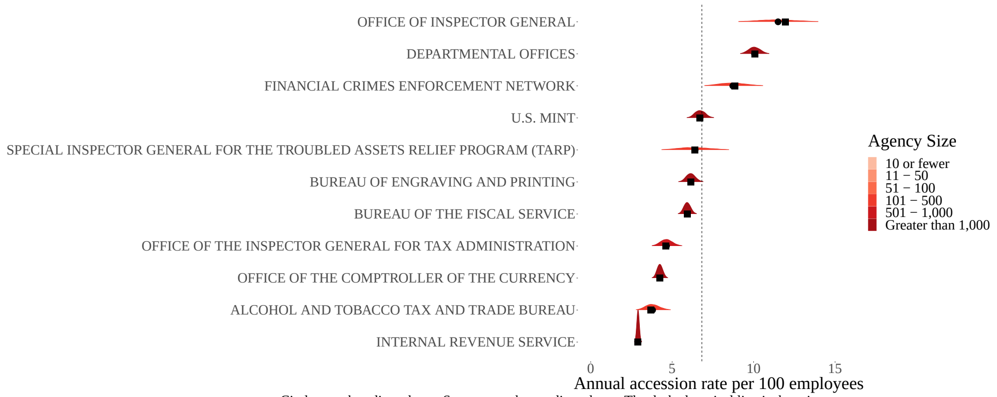

Circles are the adjusted rate. Squares are the unadjusted rate. The dashed vertical line is the prior mean.

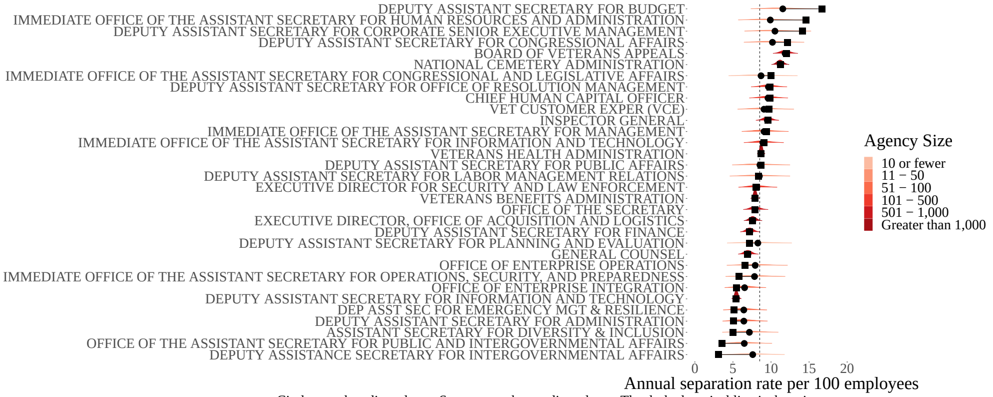

Circles are the adjusted rate. Squares are the unadjusted rate. The dashed vertical line is the prior mean.

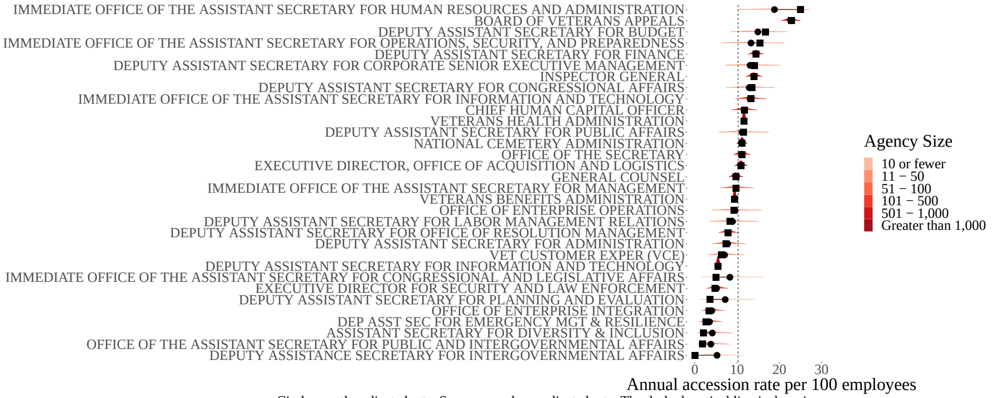

Circles are the adjusted rate. Squares are the unadjusted rate. The dashed vertical line is the prior mean.
